# Supplementary material for: CRISPR-Cas9 correction of OPA1 c.1334G>A: p.R445H restores mitochondrial homeostasis in dominant optic atrophy patient-derived iPSCs
Source: Mol Ther Nucleic Acids. 2021 Aug 19;26:432–43. doi: 10.1016/j.omtn.2021.08.015 (PMC8455316; doi:10.1016/j.omtn.2021.08.015)
Supplement: Document S1. Figures S1–S3 and Tables S1–S7 [file mmc1.pdf]

OMTN, Volume *26*

Supplemental information

CRISPR-Cas9 correction of *OPA1* c.1334G>A: p.R445H restores mitochondrial homeostasis in dominant optic atrophy patient-derived iPSCs

Paul E. Sladen, Pedro R.L. Perdigão, Grace Salsbury, Tatiana Novoselova, Jacqueline van der Spuy, J. Paul Chapple, Patrick Yu-Wai-Man, and Michael E. Cheetham

Supplementary material

CRISPR/Cas9 correction of *OPA1* c.1334G>A: p.R445H restores mitochondrial homeostasis in Dominant Optic Atrophy patient-derived iPSCs.

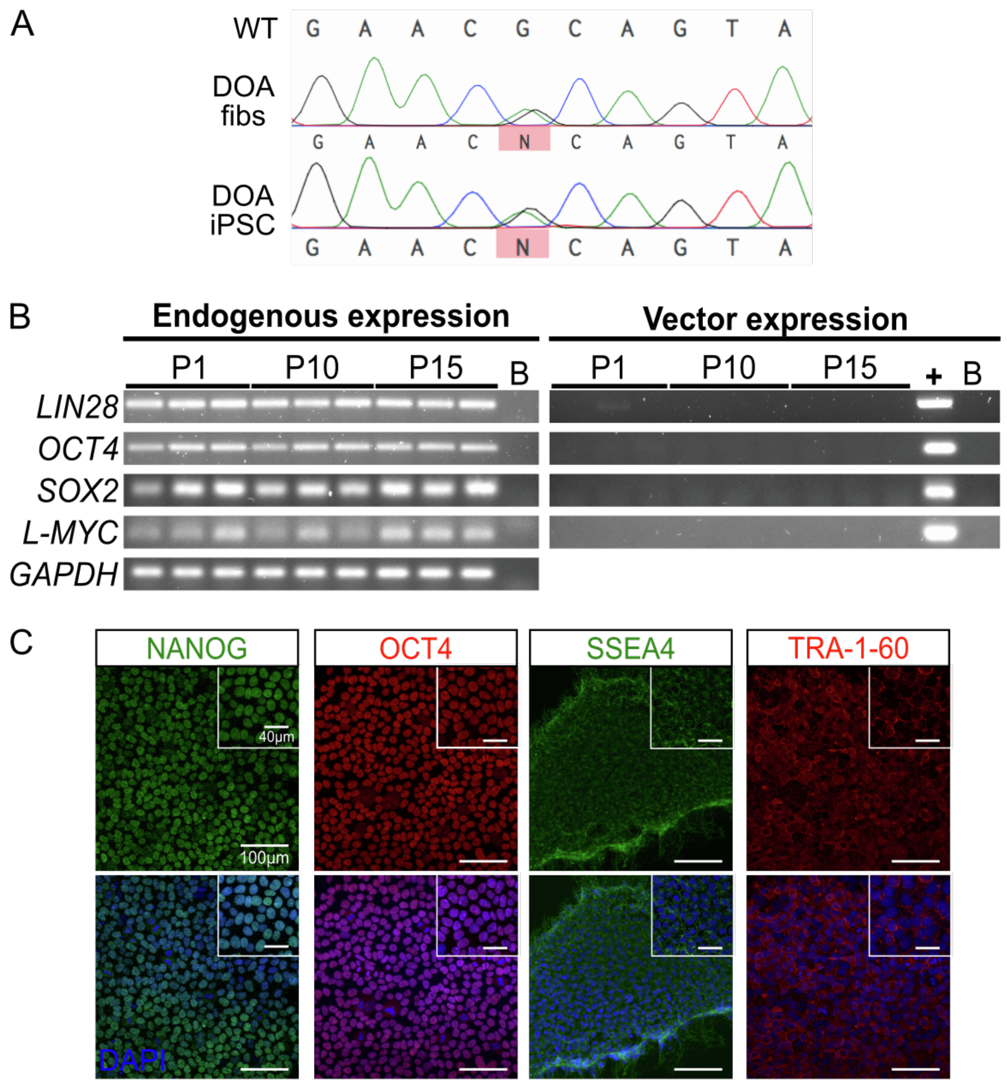

Figure S1. Generation of iPSCs carrying an *OPA1* c.1334G>A variant. Patient-derived fibroblasts carrying a c.1334G>A variant in *OPA1* were reprogrammed to iPSC by nucleofection of episomal plasmids.
(A) Post-reprogramming Sanger sequencing confirmed the presence of the G>A (N base highlighted in red) mutation within both patient-derived fibroblasts and iPSCs.
(B) RT-PCR analysis confirmed the expression of genes associated with endogenous self-renewal, including *LIN28*, *OCT4*, *SOX2*, and *L-MYC*, in DOA-iPSC. Similarly, there was negligible expression of episomal plasmid (vector) associated genes across all passage (P1, P10 and P15) of assessed iPSCs. *GAPDH* cDNA loading control. + = episomal plasmid control, B = no cDNA.
(C) Immunofluorescent staining of DOA-iPSC confirmed expression of embryonic stem cell associated proteins NANOG, OCT4, SSEA4 and TRA-160. Scale bars 100 μm and 40 μm for inset.

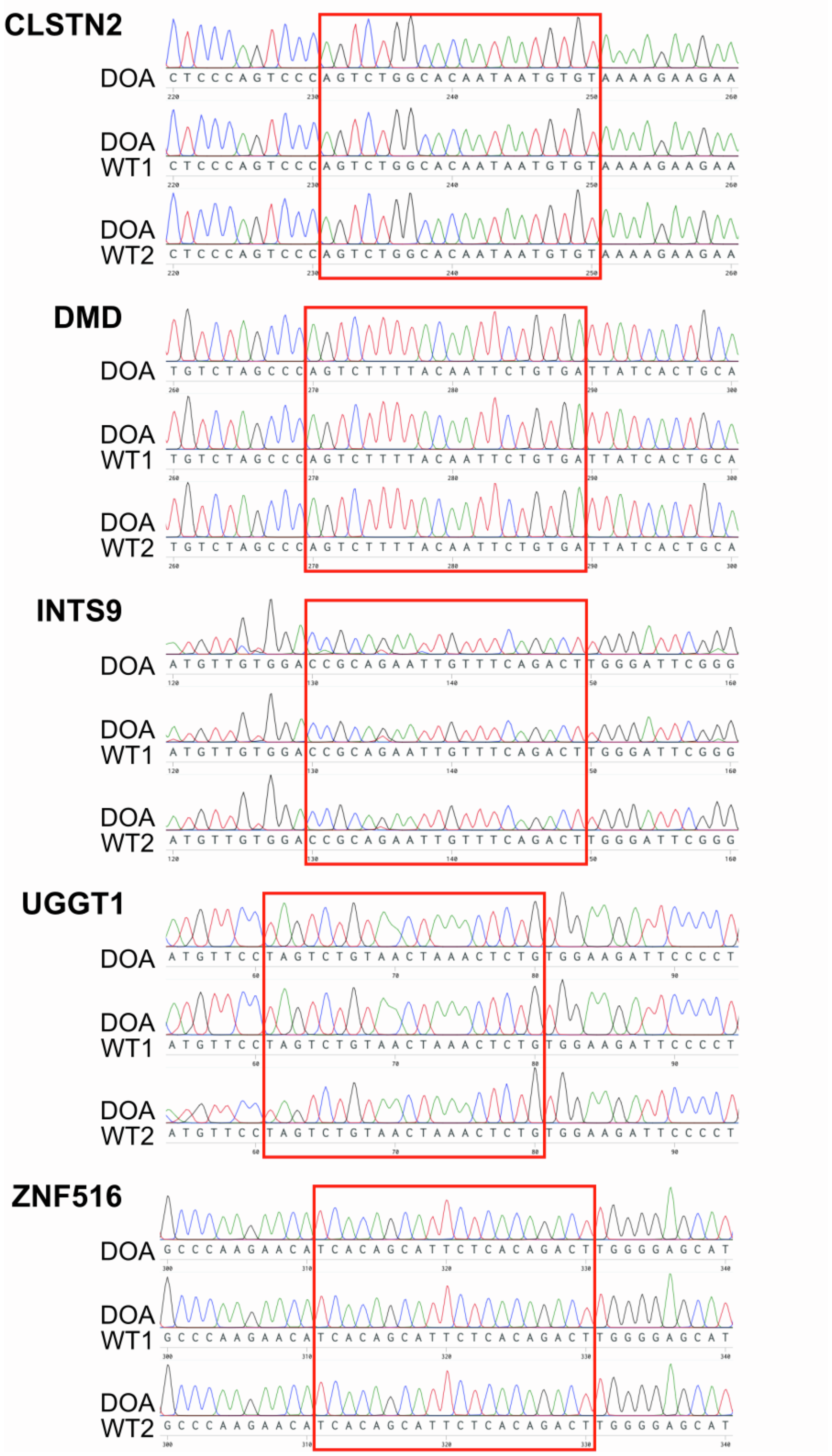

Figure S2. CRISPR/Cas9 off-target sequencing.
The top 5 predicted CRISPR/Cas9 off-target sites were determined using Off-spotter. Targets were amplified by PCR for both DOA- and corrected WT1 and WT2 iPSCs, followed by Sanger sequencing and alignment on Benchling. No mutations were found in either corrected cell line.

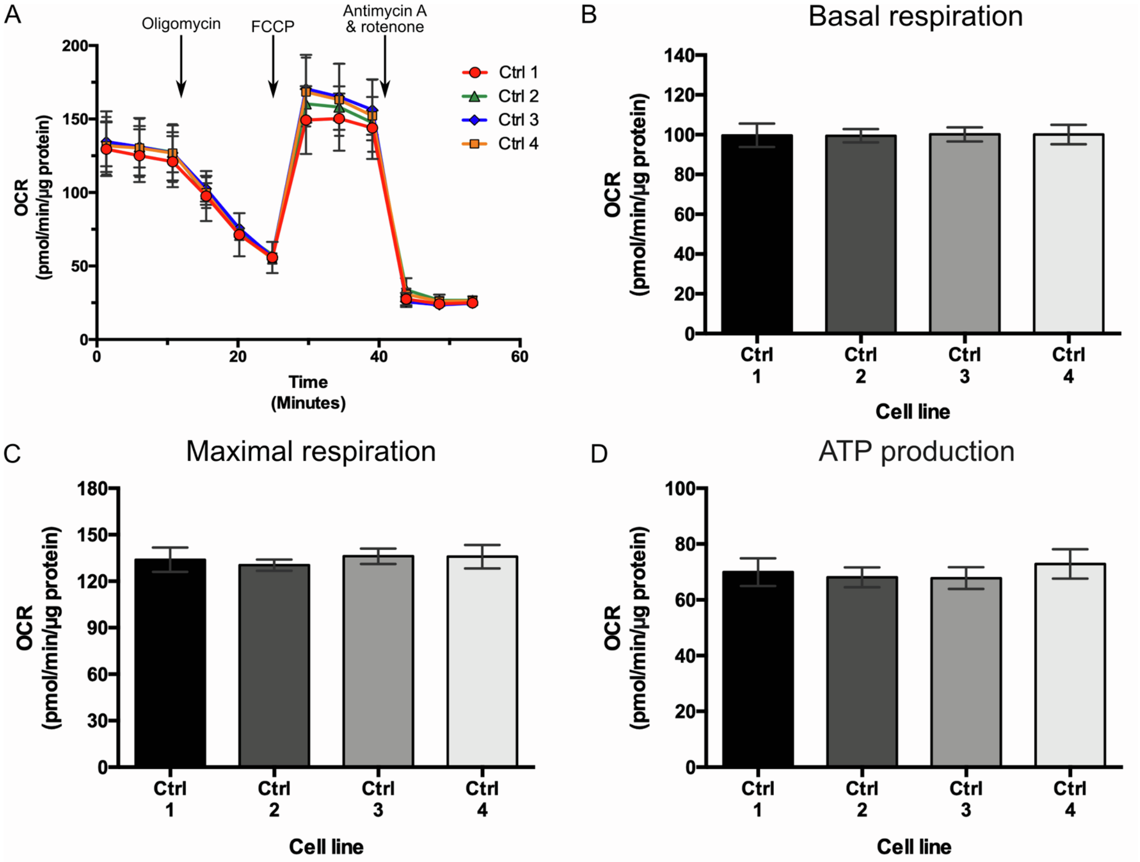

Figure S3. Comparison of mitochondrial bioenergetics for 4 independent WT iPSC lines. **(A)** Seahorse oxygen consumption rate (OCR) profiles were generated using the Seahorse XFe96 Analyzer for four independent control (ctrl) iPSC lines. 1µM oligomycin, 1µM FCCP and 0.5 µM antimycin A and 0.5 µM rotenone were injected at designated time points. Symbols represent mean OCR ± SEM.

(B-D) Analysis of bioenergetic profiles reveals the respiratory phenotype of iPSC lines. No significant differences were detected for the four independent iPSC lines for basal respiration **(B)**, maximal respiration **(C)** and ATP production **(D)**. N=34-48 replicates per cell line, from 3 separate experiments.

Table S1. Top 5 predicted off-target genes for 1334G>A gRNA 2.

The top 5 off-target genes were determined on Off-Spotter and amplified by PCR before Sanger sequencing and sequence alignment. No off-target CRISPR/Cas9 induced mutations were identified.

Gene	Chromosome	Position	gRNA mismatches	CRISPR-induced mutation
CLSTN2	3	Intronic	3	No
DMD	X	Intronic	5	No
INTS9	8	Intronic	4	No
UGGT1	2	Intronic	3	No
ZNF516	18	Intronic	5	No

Table S2. Primers used for sequencing DOA iPSCs and generation of TIDER reference template by overlap PCR.

Gene	Primer		Product size
	Forward	Reverse	
OPA1 ex 14	GCATTTTTGCCTTCTCTTCGT	TGCTTTCCTTTTGGGAGTATG	732
OPA1 ex 14 gRNA 1 C	-	GCGTTCAGCATCGACAGATCCATC	268
OPA1 ex 14 gRNA 1 D	GATGGATCTGTCGATGCTGA ACGCAGTATTGTTAC	-	488
OPA1 ex 14 gRNA 2 C	-	GTCCATTTGACTGACAAGGTCTGTA ACAATACTGCGTTCAGCATCCACAG ATCC	301
OPA1 ex 14 gRNA 2 D	TTGTTACAGACCTTGTCAGTC AAATGGAC	-	260

Table S3. gRNAs designed for targeting OPA1 exon 14.

gRNA	Guide oligonucleotide sequence	GC (%)	Distance from mutation (bp)	Predicted off-targets (Off-spotter)
gRNA 1	ATTTTTTTCAGATGGATCTG	30	65	618
gRNA 2	ACACAGTATTGTTACAGACT	35	44	314

Table S4. CRISPR/Cas9 gRNA off-target primers.

Gene	Primer		Product size
	Forward	Reverse	
CLTSN2	CTTGCCTTACAGGGTTGTGG T	GCCTCCCTGACATGACTTCTC	569
DMD	ACCATCTTCATTCCCTCTCCA	AGCCTCCACGTAAGTTGCATT	511
INTS9	AACCTGTAGACCCCTTTGCC	TCTGTGAGGGAGGAAGAAGCC	501
UGGT1	ACATGCTAACAGATGCCCAG	TCCCAACTCCCACTGAACAA	458
ZNF516	TAGGTGTGTAGGCGATGGTC	TCCTCCTGTACTAAGTGTGCAA	628

Table S5. Primers used for analysis of endogenous and vector-encoded self-renewal markers by RT-PCR.

Gene	Primer		Product size
	Forward	Reverse	
LIN29 CDS	AGCCATATGGTAGCCTCATGTCCGC	TCAATTCTGTGCCTCCGGGAGCAGGGTAGG	129
LIN28 plasmid	AGCCATATGGTAGCCTCATGTCCGC	TAGCGTAAAAGGAGCAACATAG	251
L-MYC CDS	GCGAACCCAAGACCCAGGCCTGCTCC	CAGGGGGTCTGCTCGCACCGTGATG	143
L-MYC plasmid	GGCTGAGAAGAGGATGGCTAC	TTTGTTTGACAGGAGCGACAAT	122
OCT4 CDS	CCCCAGGGCCCCATTTTGGTACC	ACCTCAGTTTGAATGCATGGGAGAGC	143
OCT4 plasmid	CATTCAAACTGAGGTAAGGG	TAGCGTAAAAGGAGCAACATAG	124
SOX2 CDS	TTCACATGTCCCAGCACTACCAGA	TCACATGTGTGAGAGGGGCAGTGTGC	80
SOX2 plasmid	TTCACATGTCCCAGCACTACCAGA	TTTGTTTGACAGGAGCGACAAT	111

Table S6. Primers used for qPCR quantification of *OPA1* expression.

Gene	Primer		Product size
	Forward	Reverse	
ACTIN	CCAACCGCGAGAAGATGA	CCAGAGGCGTACAGGGATAG	97
GAPDH	CCCCACCACACTGAATCTCC	GGTACTTTATTGATGGTACATGACAAG	105
OPA1	CGACCCCAATTAAGGACATCC	GCGAGGCTGGTAGCCATATTT	102

Table S7. Primers used to expand mtDNA via LR-PCR.

Gene	Primer		Product size
	Forward	Reverse	
mtDNA 10kb	CCCTCTCTCCTACTCCTG	CAGGTGGTCAAGTATTTATGG	9932

Table S8. Primers used to for mtDNA quantification by qPCR.

Gene	Primer		Product size
	Forward	Reverse	
B2M	CACTGAAAAAGATGAGTATGCC	AACATTCCCTGACAATCCC	231
GAPDH	CTCATCCAAGACTGGCTCCTCC	CAGCGTACTCCCCACATCAC	243
MTND1	ACGCCATAAAACTCTTCACCAAAG	GGGTTCATAGTAGAAGAGCGATGG	111
MTND4	ACCTTGGCTATCATCACCCGAT	AGTGCGATGAGTAGGGGAAGG	107
