# Supplementary material for: CRISPR-Cas9 correction of OPA1 c.1334G>A: p.R445H restores mitochondrial homeostasis in dominant optic atrophy patient-derived iPSCs
Source: Mol Ther Nucleic Acids. 2021 Aug 19;26:432–43. doi: 10.1016/j.omtn.2021.08.015 (PMC8455316; doi:10.1016/j.omtn.2021.08.015)
Supplement: Document S2. Article plus supplemental information [file mmc2.pdf]

Molecular Therapy
Nucleic Acids
Original Article

CRISPR-Cas9 correction of *OPA1* c.1334G>A: p.R445H restores mitochondrial homeostasis in dominant optic atrophy patient-derived iPSCs

Paul E. Sladen,[1] Pedro R.L. Perdigão,[1] Grace Salsbury,[2] Tatiana Novoselova,[2] Jacqueline van der Spuy,[1] J. Paul Chapple,[2] Patrick Yu-Wai-Man,[1,3,4,5] and Michael E. Cheetham[1]

[1]UCL Institute of Ophthalmology, 11-43 Bath Street, London EC1V 9EL, UK; [2]Centre for Endocrinology, William Harvey Research Institute, Barts and the London School of Medicine and Dentistry, Queen Mary University of London, Charterhouse Square, London EC1M 6BQ, UK; [3]Moorfields Eye Hospital NHS Foundation Trust, London EC1V 2PD, UK; [4]Cambridge Centre for Brain Repair, Department of Clinical Neurosciences, University of Cambridge, ED Adrian Building, Robinson Way, Cambridge CB2 0PY, UK; [5]John van Geest Centre for Brain Repair and MRC Mitochondrial Biology Unit, Department of Clinical Neurosciences, University of Cambridge, Cambridge CB2 0PY, UK

Autosomal dominant optic atrophy (DOA) is the most common inherited optic neuropathy in the United Kingdom. DOA has an insidious onset in early childhood, typically presenting with bilateral, central visual loss caused by the preferential loss of retinal ganglion cells. 60%–70% of genetically confirmed DOA cases are associated with variants in *OPA1*, a ubiquitously expressed GTPase that regulates mitochondrial homeostasis through coordination of inner membrane fusion, maintenance of cristae structure, and regulation of bioenergetic output. Whether genetic correction of *OPA1* pathogenic variants can alleviate disease-associated phenotypes remains unknown. Here, we demonstrate generation of patient-derived *OPA1* c.1334G>A: p.R445H mutant induced pluripotent stem cells (iPSCs), followed by correction of *OPA1* through CRISPR-Cas9-guided homology-directed repair (HDR) and evaluate the effect of *OPA1* correction on mitochondrial homeostasis. CRISPR-Cas9 gene editing demonstrated an efficient method of *OPA1* correction, with successful gene correction in 57% of isolated iPSCs. Correction of *OPA1* restored mitochondrial homeostasis, re-establishing the mitochondrial network and basal respiration and ATP production levels. In addition, correction of *OPA1* re-established the levels of wild-type (WT) mitochondrial DNA (mtDNA) and reduced susceptibility to apoptotic stimuli. These data demonstrate that nuclear gene correction can restore mitochondrial homeostasis and improve mtDNA integrity in DOA patient-derived cells carrying an *OPA1* variant.

INTRODUCTION

Autosomal dominant optic atrophy (DOA) is the most prevalent inherited optic neuropathy within the United Kingdom, affecting approximately 1 in 25,000 people.[1] DOA has an insidious onset during the first two decades of life, initially presenting as bilateral central vision loss, dyschromatopsia, and optic disc pallor due to gradual retinal ganglion cell (RGC) loss and optic nerve degeneration.[2,3] 60%–70% of genetically confirmed cases of DOA are caused by pathogenic variants in *OPA1*, which encodes a ubiquitously expressed GTPase protein localizing to the inner mitochondrial membrane (IMM).[1] Over 250 known pathogenic variants in *OPA1* have been reported,[4] with missense variants within the GTPase domain associated with an increased susceptibility to developing DOA plus (DOA+), a multisystemic syndromic form of the disease with extraocular features, such as sensorineural hearing loss and ataxia in addition to visual failure,[2] possibly due to a dominant-negative effect.[5] However, it remains unclear why RGCs are preferentially vulnerable to reduced OPA1 function.

OPA1 functions as a mitochondrial pro-fusion protein that regulates fusion of the mitochondrial network and the cristae morphology of the IMM.[6] Unsurprisingly, *OPA1* disease-associated variants are associated with an increased fragmentation of the mitochondrial network and disruption of the IMM structure.[7–9] Generation of DOA mouse models through targeted *Opa1* disruption, resulting in truncation of OPA1 and haploinsufficiency, further confirmed the effects seen in cell models.[6,10] OPA1 is also an important regulator of cellular bioenergetic output,[11] with evidence demonstrating that loss of OPA1 function results in reduced bioenergetic output, including reduced levels of adenosine triphosphate (ATP) synthesis.[8,9,12] Bioenergetic reduction is likely due to mitochondrial respiratory chain complex instability, primarily of complex I and ATP synthase, reduced efficiency of mitochondrial electron flux, and disturbed cristae structure.[12] Recently it has been demonstrated

Received 28 October 2020; accepted 9 August 2021;
https://doi.org/10.1016/j.omtn.2021.08.015.

Correspondence: Michael E. Cheetham, UCL Institute of Ophthalmology, 11-43 Bath Street, London EC1V 9EL, UK.
E-mail: michael.cheetham@ucl.ac.uk
Correspondence: Patrick Yu-Wai-Man, Cambridge Centre for Brain Repair, Department of Clinical Neurosciences, University of Cambridge, ED Adrian Building, Robinson Way, Cambridge CB2 0PY, UK.
E-mail: py237@cam.ac.uk

 © 2021 The Author(s).
This is an open access article under the CC BY license (http://creativecommons.org/licenses/by/4.0/).

that all 8 splice isoforms of human *OPA1* are capable of rescuing ATP synthesis within *Opa1* null mouse embryonic fibroblasts (MEFs), independent of their ability to rescue mitochondrial morphology, suggesting that functional fusion alone is not essential for OPA1-mediated regulation of energy output.[13]

OPA1 is also important for maintaining mitochondrial DNA (mtDNA) integrity. OPA1 interacts with mtDNA nucleoids through exon 4b binding to mtDNA D-loops, and decreased expression of exon 4b isoforms significantly reduced mtDNA copy number and inhibited mtDNA distribution throughout the mitochondrial network.[13,14] Furthermore, DOA patient cells exhibit reduced mtDNA copy number[12,15,16] and accumulate somatic mtDNA mutations.[16–19] Given that mtDNA encodes for key components of the respiratory chain, *OPA1* variants exert a significant detrimental effect on mitochondrial biogenesis and mtDNA stability. In addition, reduced OPA1 function has been shown to increase cellular susceptibility to apoptotic stimuli,[12,20] predisposing cells to intrinsic and extrinsic toxic insults.

Current therapeutic options to treat DOA are limited. Idebenone, a ubiquinone analog, has been shown to protect against visual loss in Leber hereditary optic neuropathy (LHON), a clinically related condition also characterized by preferential RGC loss.[21] Retrospective off-label clinical trials in *OPA1*-related DOA patients have suggested some potential benefit of idebenone, with treated patients demonstrating recovery of visual parameters compared to untreated patients;[22] however, a placebo-controlled trial in an *Opa1* mouse model showed only a transient benefit of idebenone treatment.[23] Gene therapy presents a promising option, as the majority of *OPA1*-related DOA is associated with haploinsufficiency.[24] Expression of human *OPA1* under the CMV promoter in an *Opa1* mouse model increased RGC survival and improved visual function.[25] However, there is concern that OPA1 expression must be carefully controlled, as too much OPA1 may have detrimental effects.[26] Recent evidence from a gene therapy study in LHON patients demonstrated significant bilateral visual improvements, demonstrating the feasibility of gene therapy for treating inherited optic neuropathies.[27,28]

The opportunity to correct the genetic mutation *in situ* presents an exciting therapeutic prospect. CRISPR-Cas9 technology revolutionized the gene editing field, enabling rapid and efficient correction of genetic mutations through a variety of techniques, such as homology-directed repair (HDR).[29] CRISPR-Cas9 gene editing provides an opportunity for *in vitro* correction of patient-derived cells, generating isogenic cell lines to facilitate and enhance our knowledge of pathogenic mutation disease mechanisms. To date, there are no reports on the correction of *OPA1* or the effect of genetic correction on mutant-associated phenotypes. Here, we describe the CRISPR-Cas9 correction of a patient-derived *OPA1* induced pluripotent stem cell (iPSC) line and show that nuclear gene correction can restore mitochondrial homeostasis, including mtDNA stability and cellular bioenergetics.

RESULTS

Generation of *OPA1* mutant iPSCs and isogenic controls via CRISPR-Cas9

To establish and characterize a human DOA disease model, dermal fibroblasts from an affected individual with DOA harboring a missense variant c.1334G>A: p.R445H (DOA-iPSC), which is associated with DOA+, in the *OPA1* GTPase domain, were re-programmed to iPSCs alongside wild-type (WT) control fibroblasts using non-integrating episomal plasmids and nucleofection.[30] The presence of the heterozygous *OPA1* missense variant was confirmed in DOA-iPSCs (Figure S1A). RT-PCR analysis of DOA-iPSC confirmed the induction of endogenous self-renewal genes *OCT4*, *L-MYC*, *SOX2*, and *LIN28*, while episomal plasmid expression was negligible at passage 1 (see Lin28 P1 lane 2) and undetectable by passages 10 and 15 (Figure S1B), confirming the loss of episomal plasmids. To investigate pluripotency, DOA-iPSCs were analyzed via immunofluorescence (IF) staining for the presence of embryonic stem cell markers. IF analysis confirmed protein expression of OCT4 and NANOG, localized to the cell nucleus, and cell surface expression of both SSEA4 and TRA-1-60 (Figure S1C).

Following their generation, DOA-iPSCs were corrected using CRISPR-Cas9 gene editing technology combined with a single-stranded DNA (ssDNA) repair template.[31,32] Two CRISPR-Cas9 guides were designed to direct the Cas9 endonuclease to cut as close as possible to the *OPA1* c.1334G>A variant (Figure 1A), cutting 15 bp upstream and 14 bp downstream of the point variant, respectively. Both guide RNAs (gRNAs) targeting the *OPA1* c.1334G>A mutation were selected using a web-based software tool (Benchling), which enables the design of optimal gRNAs through algorithms to predict on-target and off-target activity.[33,34] ssDNA templates were designed to asymmetrically overlap the Cas9-induced dsDNA break site and to correct the A>G variant at position c.1334 while inducing synonymous changes at the Sp.Cas9 gRNA protospacer-adjacent motif (PAM) site required for Cas9 activity, thus preventing further cleavage of *OPA1* (Figure 1A). To validate the specificity of both gRNAs, iPSCs were nucleofected with ribonucleotide protein (RNP) complexes containing the *OPA1*-targeting gRNAs or non-targeting gRNAs. Genomic DNA (gDNA) was subsequently extracted and a T7EI assay was completed, demonstrating that both *OPA1* gRNAs induced gene editing events within the target region, whereas both non-targeting gRNAs failed to do so (Figure 1B).

Subsequently, DOA-iPSCs were nucleofected with RNP complexes and the corresponding ssDNA templates. iPSCs were allowed to expand before analysis of gDNA extracted from pooled single cells was used to determine CRISPR-Cas9-mediated *OPA1* editing and correction efficiency through TIDER analysis. Sanger sequencing traces of pooled, edited gDNA were uploaded into the TIDER online algorithm to estimate the frequency of total RNP cutting efficiencies and HDR editing in all transfection conditions.[35] This analysis confirmed that both gRNAs promoted *OPA1* gene editing, demonstrating the total RNP cutting efficiency, an estimation of all HDR and non-homologous end joining (NHEJ) events induced by the

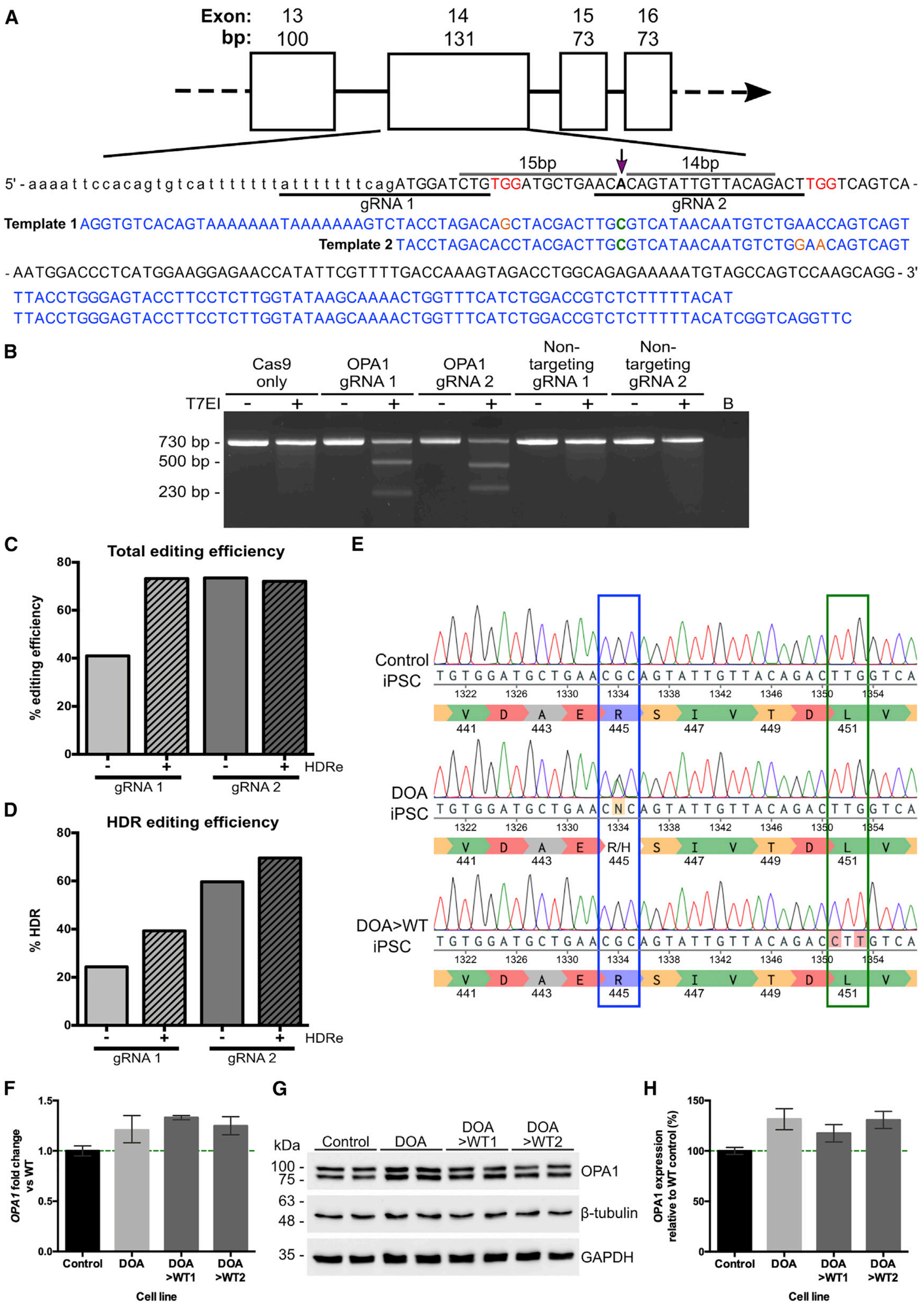

(legend on next page)

targeted dsDNA breaks, for gRNA 2 (73%) was significantly higher than gRNA 1 (41%; Figure 1C). However, HDR enhancer (HDRe) significantly improved the editing efficiency of gRNA 1, up to 73%, whereas it had little to no effect on gRNA 2 (Figure 1C). Analysis of the HDR editing efficiencies supported these findings, demonstrating an HDR editing of 24% for gRNA 1, while gRNA 2 exhibited an editing efficiency of approximately 60% (Figure 1D). Again, both gRNAs exhibited increased editing efficiency with the addition of HDRe, an increase to 40% and 70% HDR efficiency for gRNA 1 and 2, respectively (Figure 1D). Therefore, iPSCs transfected with both gRNA 2 and template 2, with and without HDRe, were selected for further clonal isolation due to the significantly higher levels of successful HDR editing.

Individual iPSC clones were manually isolated and cultured for a further 7 days before Sanger sequencing. Correctly edited clones were identified by G homozygosity at position c.1334 and introduction of synonymous changes at the PAM site (Figure 1E). Following correction of the c.1334G>A variant, quantitative PCR (qPCR) analysis was utilized to determine the effect of gene correction on *OPA1* expression. Both independent DOA>WT iPSC lines showed similar levels of *OPA1* expression compared to DOA-iPSCs, which was a moderate, non-significant increase when compared to WT control iPSCs (Figure 1F). This suggests the 1334G>A variant is not driving elevated levels of *OPA1* transcript in the DOA-iPSCs. Subsequently, western blot analysis for OPA1 protein showed a non-significant increase in OPA1 levels for both DOA and DOA>WT iPSCs when compared to endogenous WT cells (Figures 1G and 1H), correlating with the increase observed in *OPA1* mRNA and suggesting that the R445H amino acid substitution does not affect protein level.

Finally, to determine if CRISPR-Cas9 editing introduced off-target effects, gRNA 2 target sequence was analyzed via the Off-Spotter program for regions with high sequence-homology to the gRNA target. The top 5 potential off-target sites were determined (Table S1) and amplified through PCR for both DOA-iPSCs and corrected DOA-WT iPSCs. PCR products were Sanger sequenced and aligned on Benchling. Edited iPSC lines showed no evidence of off-target mutations when compared to unedited DOA-iPSCs (Figure S2; Table S1).

Gene correction of *OPA1* rescues mitochondrial network structure

We subsequently investigated if CRISPR-Cas9 gene correction could restore mitochondrial network morphology using an endogenous control iPSC line, along with non-edited isogenic DOA-iPSCs and two independently isolated *OPA1* c.1334G>A-corrected DOA-iPSC clones (termed DOA>WT1 and DOA>WT2). Confocal imaging of mitochondrial networks stained with an anti-TOMM20 antibody demonstrated significant levels of mitochondrial fragmentation in DOA-iPSCs when compared with endogenous WT control cells and DOA>WT iPSCs (Figure 2A). Analysis of mitochondrial networks using Mitochondrial Network Analysis (MiNa),[36] a semi-automated ImageJ plugin, enabled quantification of network parameters, demonstrating significantly higher levels of individual mitochondria and mitochondrial networks for DOA-iPSCs when compared to both DOA>WT or control iPSCs (Figure 2B), indicative of greater mitochondrial fragmentation. In addition, DOA-iPSCs demonstrated significantly reduced levels of network branches (Figure 2C) and significantly shorter network branch length (Figure 2D) when compared to both control and DOA>WT iPSCs. These data suggest gene correction has restored most of the DOA mitochondrial network parameters to control cell levels. However, DOA>WT iPSCs had significantly higher levels of mitochondrial branches than the control cells, but these were significantly shorter (Figures 2C and 2D), suggesting a network that has greater connectivity but with slightly shorter branches. These differences could reflect physiological cell line variations in otherwise healthy network parameters.

CRISPR-Cas9 correction of *OPA1* restores mitochondrial bioenergetic function

To determine if correction of DOA-iPSCs had downstream effects on mitochondrial function, four WT control iPSC lines from independent individuals, along with DOA-iPSCs and DOA>WT iPSC lines were analyzed by Seahorse technology to determine rescue of

Figure 1. CRISPR-Cas9-directed gene correction of *OPA1* c.1334G>A in DOA patient-derived iPSCs

(A) Design of *OPA1*-targeting gRNA and ssDNA template for correction of c.1334G>A by homology-directed repair (HDR). Two gRNAs—gRNA1 and gRNA2—were designed to respectively cut 15 bp upstream and 14 bp downstream of the target mutation (purple arrow). For each gRNA, complementary ssDNA HDR templates (blue) were designed to correct A>G base change (green) with asymmetric homology arms of 36 and 91 nucleotides relative to the gRNA cutting position. Each ssDNA template included synonymous base changes (orange) to remove the corresponding gRNA PAM site and prevent re-cutting following recombination. (B) Validation of *OPA1* gRNA specificity via T7 endonuclease I assay. DOA-iPSCs were treated with Cas9 only, each *OPA1* gRNA RNPs, or non-targeting gRNA RNPs. The target site surrounding the c.1334 mutation was expanded and treated with T7EI, heteroduplexes formed, and subsequent resolution by gel electrophoresis. Both *OPA1* gRNA RNPs demonstrate significant levels of gDNA cleavage when compared to Cas9 only or non-targeting gRNA RNPs. (C) TIDER estimation of total *OPA1*-targeting CRISPR-Cas9 cutting efficiency, in the presence or absence of 30 μM HDR enhancer (HDRe), by analyzing the frequency of induced insertions or deletions (indels) out of the total RNP-induced base changes. (D) TIDER analysis estimate of HDR efficiency in the absence or presence of 30 μM HDRe. Analysis determines the percentage of sequences containing the desired ssDNA-induced base changes. (E) Alignment of Sanger sequencing traces of WT iPSC with isogenic lines of DOA-patient iPSC and HDR edited (DOA>WT) clones on Benchling. Correctly edited DOA>WT clones were identified through G homozygosity at position 1,334 (blue box) and homozygous synonymous PAM site changes (green box). (F) qPCR analysis of DOA-iPSCs and CRISPR-Cas9 gene-corrected DOA>WT1 and >WT2 iPSCs showed moderately increased *OPA1* expression when compared to endogenous WT *OPA1* iPSCs. *OPA1* expression was first normalized to the geometric mean of reference genes *GAPDH* and *ACTIN*, and then to WT iPSC expression. (G) Representative western blot analysis of OPA1 expression in WT control, DOA, and DOA>WT iPSCs. (H) Quantification of OPA1 expression in WT control, DOA, and DOA>WT iPSCs. OPA1 expression was first normalized to GAPDH expression and then to WT control expression. Bars represent average expression relative to the WT control ± standard error of the mean (SEM).

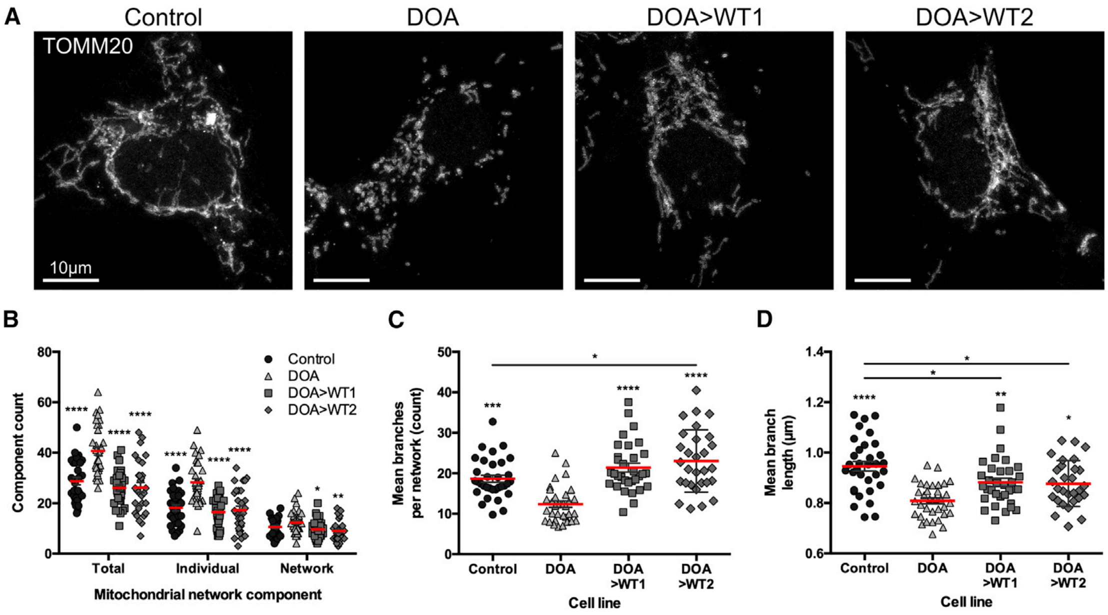

Figure 2. Mitochondrial network analysis in DOA-iPSC and CRISPR-Cas9-corrected DOA-WT iPSCs

(A) Representative confocal images of control, DOA, and DOA>WT iPSCs stained with anti-TOMM20 to identify mitochondrial networks. Scale bars represent 10 μm. (B) Quantification of the mitochondrial network composition, determined as total components, individual mitochondria (mitochondria that have no branches), and mitochondrial networks (interconnected or branched mitochondria). (C) Mean branches per mitochondrial network (counts). (D) Mean network branch length, the distance between two branch points in a mitochondrial network. Red bar denotes mean ± SEM. n = minimum 30 individual iPSCs from at least 3 separate cell preparations. *$p < 0.05$, **$p < 0.005$, ***$p < 0.001$, ****$p < 0.0001$ versus DOA iPSCs or indicated cell lines.

mitochondrial respiratory chain function. Seahorse bioenergetic oxygen consumption rate (OCR) profiles were generated for WT control lines, DOA-iPSC, and DOA>WT1 and DOA>WT2 iPSCs (Figure 3A). Injection of compounds targeting the mitochondrial respiratory transport chain caused alterations to OCR levels: oligomycin caused reduced levels of OCR, carbonyl cyanide p-trifluoromethoxyphenylhydrazone (FCCP) uncoupled the respiratory transport chain causing a rapid increase of OCR, and, finally, antimycin A and rotenone injection caused a reduction in OCR level (Figure 4A).

Comparison of four independent control cell lines demonstrated no significant differences for basal respiration, maximal respiration, and ATP production (Figure S3); as such, subsequent analysis utilized grouped control data. Analysis of bioenergetic profiles demonstrated significant differences between the DOA-iPSCs and control cell lines. DOA-iPSCs exhibited significantly reduced basal respiratory rate when compared to both WT control lines and corrected DOA>WT1 and DOA>WT2 cell lines, both of which were similar to WT control lines (Figure 3B). Similarly, DOA-iPSC had a significantly reduced level of maximal respiration versus control and corrected DOA>WT cell lines (Figure 3C). Interestingly, the level of maximal respiration for one DOA>WT cell line was significantly higher than the grouped control iPSCs (Figure 3C). Analysis of ATP production demonstrated significantly reduced levels of ATP production for DOA-iPSCs when compared to control iPSCs (Figure 3D), while CRISPR-Cas9 gene correction restored ATP production in both DOA>WT1 and DOA>WT2, demonstrating levels of ATP production equivalent to that of the WT iPSC lines (Figure 3D).

Correction of *OPA1* restores WT mitochondrial DNA levels

Next, we determined whether *OPA1* c.1334G>A variant impacted mtDNA integrity and analyzed if nuclear gene correction could restore mtDNA homeostasis. Initial analysis of iPSCs via long-range PCR (LR-PCR) demonstrated no clear product indicating mtDNA deletions for any iPSCs when compared to a positive control (Figure 4A). In contrast, subsequent qPCR demonstrated significant levels of mtDNA deletion in DOA-iPSCs when compared to WT control iPSCs, with a reduction to 83.8% ± 5.5% of WT mtDNA levels (Figure 4B). Interestingly, CRISPR-Cas9 gene editing of *OPA1* had a significant impact on WT mtDNA composition. Both CRISPR-corrected DOA>WT1 and DOA>WT2 iPSCs showed restored levels of WT mtDNA back to that of control cells, showing 98.5% ± 4.3% and 99.4% ± 6.7% of WT mtDNA levels, respectively, a significant increase when compared to DOA-iPSC (Figure 4B). Analysis of mtDNA copy number, defined by the ratio of *MTND1* to nuclear DNA, showed no significant differences across the 4 cell lines, suggesting the *OPA1* variant does not induce copy number changes in iPSCs (Figure 4C).

Correction of *OPA1* reduces apoptosis susceptibility associated with *OPA1* variants

Finally, the effect of CRISPR-Cas9 *OPA1* correction on the susceptibility of iPSCs to apoptotic stimuli was investigated. Control, DOA, and DOA>WT iPSCs were treated with staurosporine, an apoptosis inducer, and apoptosis was quantified via flow cytometry using Annexin V and propidium iodide (PI). After 8 h of

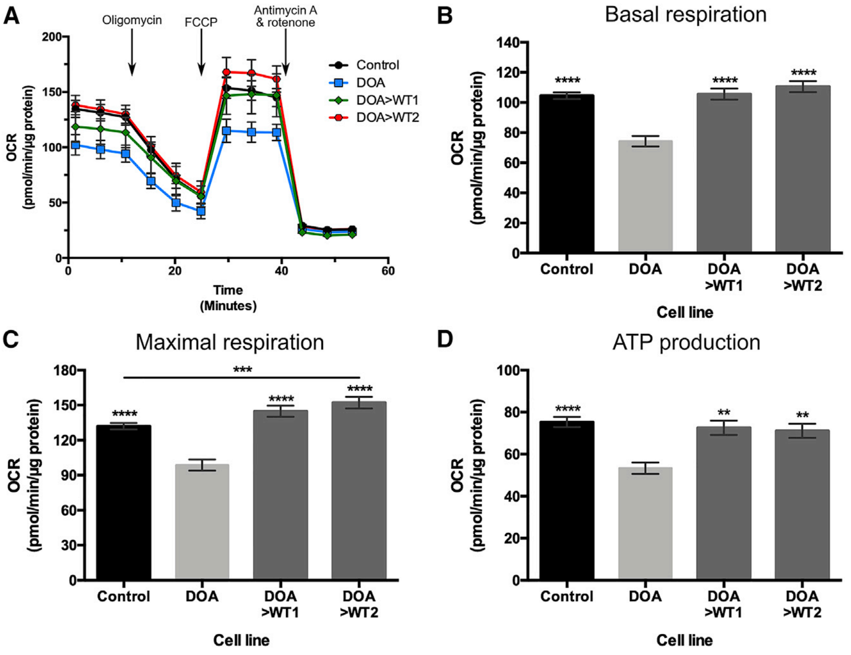

Figure 3. Seahorse analysis of DOA-iPSC and CRISPR-Cas9-corrected DOA-WT iPSCs

(A) Seahorse oxygen consumption rate (OCR) profiles were generated using the Seahorse XFe96 Analyzer for WT control (mean of 4 independent lines), DOA-iPSCs, and DOA>WT1 and DOA>WT2 iPSC lines. 1 μM oligomycin, 1 μM FCCP, and 0.5 μM antimycin A and 0.5 μM rotenone were injected at designated time points. Symbols represent mean OCR ± SEM. (B–D) Analysis of bioenergetic profiles reveals the respiratory phenotype of iPSC lines. DOA-iPSCs have lower levels of basal respiration (B), maximal respiration (C), and ATP production (D) than WT control and CRISPR-corrected DOA>WT1 and DOA>WT2 iPSC lines. n = at least 57 replicates per cell line, from a minimum of 3 separate experiments. Bars represent mean OCR ± SEM. *$p < 0.05$, ****$p < 0.0001$ versus DOA-iPSCs or indicated cell lines.

treatment, DOA-iPSCs demonstrated significantly higher levels of Annexin V-positive and PI-negative cells when compared to control and DOA>WT iPSCs (Figures 5A and 5C), indicating an increase in "early" apoptotic cells in DOA-iPSCs at this time point. By 24 h of staurosporine treatment, DOA-iPSCs demonstrated a significantly higher percentage of Annexin V-positive and PI-positive "late" apoptotic cells, when compared to control and DOA>WT iPSCs (Figures 5B and 5D). This effect was further evaluated through analysis of lactate dehydrogenase (LDH) release, a cytosolic enzyme released during cellular apoptosis. When treated with staurosporine, DOA-iPSCs demonstrated significantly higher levels of LDH release when compared to control and DOA>WT iPSCs (Figure 5E), confirming the significantly higher levels of cytotoxicity for staurosporine in DOA-iPSCs and the reduced susceptibility to apoptosis conferred by gene correction in DOA>WT iPSCs.

DISCUSSION

Despite our current knowledge surrounding the function of OPA1 in mitochondrial homeostasis, therapeutic options for DOA remain limited. The discovery of CRISPR-Cas9 gene editing technology has revolutionized the disease modeling field, enabling efficient generation of isogenic cell lines carrying disease-specific mutations. Here, we report the successful implementation of a gene editing approach for HDR-mediated correction of an *OPA1* variant in iPSCs derived from DOA patient-derived fibroblasts. DOA-iPSCs were targeted for gene correction using CRISPR-Cas9 and a ssDNA repair template to enable HDR. Analysis of treated iPSC populations demonstrated that HDR was attained with efficiencies up to 70%, enabling rapid and efficient isolation of DOA>WT iPSC clones. Importantly, variant correction had significant downstream benefits on mitochondrial homeostasis, restoring mitochondrial network fusion and bioenergetic function. In addition, functional restoration of WT mtDNA levels was observed following correction of a nuclear encoded gene. Importantly, CRISPR-Cas9 gene correction removed the susceptibility of iPSCs to apoptotic stimuli.

To date, most of the data on the multifunctional roles of OPA1 have come from the study of patient-derived fibroblasts or model cell lines.[9,13,17] Crucially, exogenous expression of *OPA1* has been shown to rescue variant-associated phenotypes, while *OPA1* gene therapy has shown promise as an effective strategy to prevent RGC loss.[13,25] Recent clinical trials utilizing allotopic gene expression to replace the mutant *ND4* gene in LHON patients carrying the m.11778G>A mtDNA mutation demonstrated significant bilateral visual improvements, further highlighting the potential of gene therapy as a promising treatment option for the inherited optic neuropathies.[27,28] However, overexpression of *OPA1* also results in mitochondrial fragmentation, and therapeutic strategies must strike a delicate balance to maintain mitochondrial homeostasis. As such, restoration of endogenous physiological levels of OPA1 function, through technologies such as CRISPR-Cas9, provide an alternative approach that capitalizes on endogenous cell machinery to regulate both total expression and alternative splicing. Importantly, this allows cells to regulate specific isoform expression to facilitate specific mitochondrial functions,[13,14] demonstrated here with restoration of bioenergetic output, the mitochondrial network, and mtDNA quality control.

Although this study provides a proof of concept in mitotic, rapidly dividing cells, which could facilitate the reduction in dysfunctional mitochondria, it further demonstrates the interconnection between both nuclear and mitochondrial genomes.[37] The protocol for gene correction took approximately 4 weeks to generate corrected patient iPSC lines; therefore, these cells went through a significant number of doublings. However, it raises the question of how quickly post-mitotic

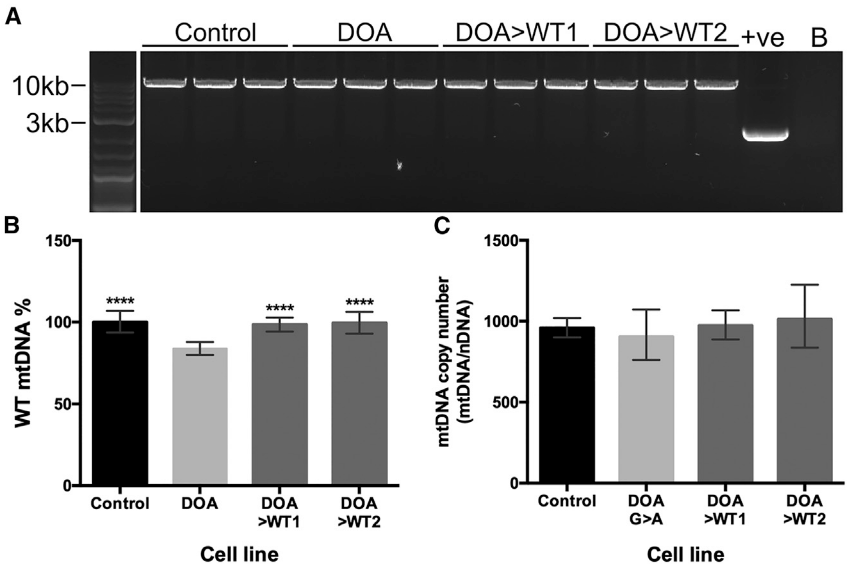

Figure 4. Analysis of iPSC mtDNA quantity and quality by LR-PCR and qPCR

(A) LR-PCR analysis of large-scale mtDNA deletions in the 4 iPSC lines. +ve, cybrid DNA with deleted mtDNA. B, no DNA control. (B) Quantification of WT mtDNA levels (%) via qPCR using mtDNA genes *MTND4* and *MTND1*. *MTND4* quantity was normalized to *MTND1* levels and then to WT control. N = 6 samples per genotype. Bars represent mean WT mtDNA % ± SEM. ****$p < 0.0001$ versus DOA-iPSCs. (C) *OPA1* mutation has no effect on mtDNA copy number in DOA-iPSCs. MtDNA copy number was quantified by the ratio of *MTND1* versus the geometric mean of the number copies of nuclear DNA genes *GAPDH* and *B2M.* n = 6 samples per cell line.

cells could restore mitochondrial homeostasis following nuclear gene correction, especially in cells with diffuse mitochondrial networks like RGCs. Sarzi and colleagues previously demonstrated improved survival of RGCs in a mouse model of DOA after 8 months of OPA1 gene therapy.[25] However, this model provided no intermediate time points for mitochondrial rescue and did not examine the effects of *OPA1* rescue on mtDNA integrity. Importantly, we have demonstrated that correction of an *OPA1* variant associated with DOA reduced sensitivity to apoptotic stimuli, suggesting that CRISPR-Cas9 gene editing of RGCs may increase their survival within the retina. However, CRISPR-Cas9 gene editing has its inherent limitations. HDR, the method utilized to correct DOA-iPSCs within this study, primarily acts during the S/G2 phases of cell division in mitotic cells.[38] It is therefore highly inefficient in post-mitotic neurons, which presents additional challenges for efficient *in vivo* gene editing of RGCs. Therefore, further research into *in vivo* gene augmentation and CRISPR-Cas9 gene correction is required to establish the plausibility for RGC gene editing and the time required for mitochondrial homeostasis restoration in post-mitotic neurons.

The mitochondrial genome encodes 37 genes that are essential for mitochondrial function.[39] Disturbed mtDNA maintenance with loss of mtDNA integrity is a pathological hallmark shared by a number of neurodegenerative phenotypes, including monogenic diseases caused by *OPA1* mutations and more complex, late-onset neurological diseases, such as Parkinson's disease.[12,15–19,40] In particular, LHON, which is caused by specific mutations within the mitochondrial genome, highlights the detrimental effects of mutated mtDNA on RGC health.[41] Thus, the ability to restore mtDNA integrity is a desirable therapeutic goal to rescue mitochondrial function. Numerous studies have now demonstrated the feasibility of selectively targeting mtDNA for gene editing, yet, mtDNA editing remains problematic due to inefficient mitochondrial nucleotide import efficiency.[42] Most recently, CRISPR-free mtDNA editing has been demonstrated utilizing a bacterial cytidine deaminase toxin that enables the precise, targeted manipulation of mtDNA.[43] In spite of this advance, due to their somatic origins, mtDNA mutations associated with DOA and other neurodegenerative conditions are heterogeneous in nature and thus impractical to selectively target for correction.[40] Therefore, the possibility of nuclear gene correction to restore WT mtDNA levels is a promising prospect for a range of neurological conditions.

Interestingly, rescue of mtDNA integrity via nuclear gene correction has previously been demonstrated following correction of *LRRK2* mutant cells via zinc-finger nucleases.[44,45] However, the exact mechanism behind this rescue remains unclear, given the typical cytosolic localization of LRRK2. Conversely, our data demonstrate the restoration of mitochondrial homeostasis through genetic correction of a canonical nuclear-encoded mitochondrial protein. Recent research has demonstrated that *OPA1* maintains mtDNA integrity through direct interaction of exon 4b and the mitochondrial D-loop, anchoring mtDNA within the mitochondrial inner membrane to facilitate replication and distribution.[14] Furthermore, expression of OPA1 mutants incapable of GTP hydrolysis does not restore mtDNA content in $Opa1^{-/-}$ mouse embryonic fibroblasts, indicating a functional GTPase domain is required for mtDNA stability.[13] Our data confirm these findings, as we identified moderate reduction of WT mtDNA in our DOA-iPSCs, which were restored upon gene correction. Furthermore, these data support the theory that GTPase point mutations produce a dominant-negative effect,[17] reducing the functional capabilities of WT OPA1 for maintaining mtDNA stability.

Overall, we have shown that correction of *OPA1* successfully rescues the pathological mitochondrial phenotypes typically associated with mutations in this nuclear gene, notably mitochondrial fragmentation, impaired mitochondrial bioenergetic output, mtDNA instability, and apoptotic susceptibility. Generation of isogenic cell lines provides an invaluable opportunity to dissect the disease mechanisms associated with specific *OPA1* variants, which will facilitate the development of targeted treatments for this currently untreatable form of inherited optic neuropathy caused by mitochondrial dysfunction.

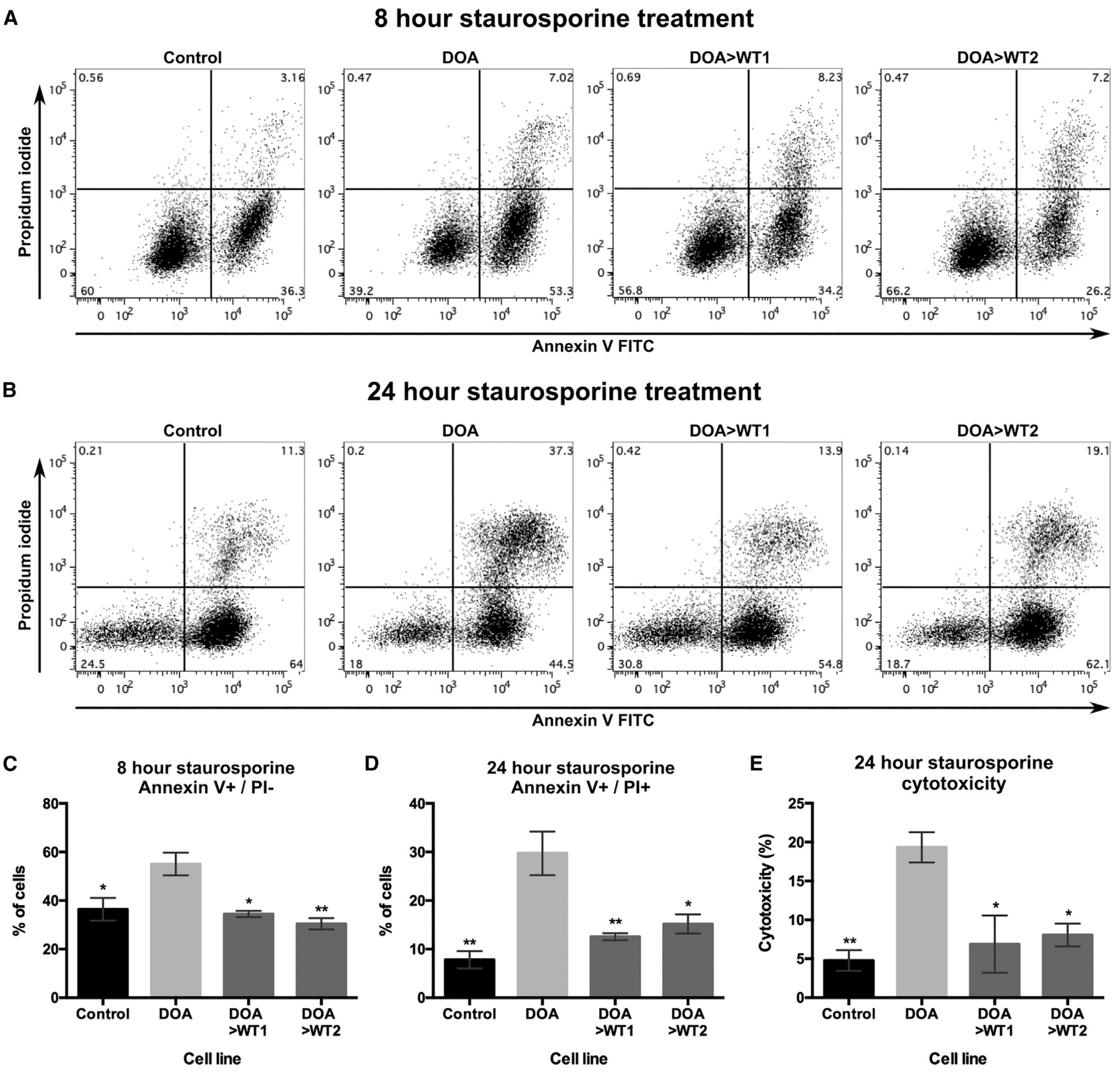

Figure 5. CRISPR-Cas9 gene correction of *OPA1* reduces susceptibility to apoptotic stimuli

(A and B) Representative flow cytometry analysis of control, DOA, and DOA>WT iPSCs following (A) 8 h, or (B) 24 h of treatment with 10 nM staurosporine. iPSCs were labeled with early apoptosis marker Annexin V and late apoptosis marker propidium iodide (PI). (C and D) Quantification of (C) early apoptotic Annexin V+/PI− and (D) late apoptotic Annexin V+/PI+ iPSCs after (C) 8 h or (D) 24 h of treatment with 10 nM staurosporine. N = 3 samples per cell line. Bars represent mean percent of Annexin V+/PI− cells ± SEM. $^{*}p < 0.05$, $^{**}p < 0.005$ versus DOA-iPSCs. (E) LDH release quantification after treatment of iPSCS for 24 h with 10 nM staurosporine, determined as percentage of cellular cytotoxicity per cell line. n = 3 samples per cell line. Bars represent mean cytotoxicity ± SEM. $^{*}p < 0.05$, $^{**}p < 0.005$ versus DOA-iPSCs.

MATERIALS AND METHODS

Cell reprogramming and CRISPR-Cas9 gene editing

Primary fibroblasts were established from a skin biopsy taken from an affected individual with DOA+ and carrying a heterozygous *OPA1* c.1334G>A: p.R445H variant. Informed consent was obtained following the tenets of the Declaration of Helsinki. Ethical approval was granted by the Yorkshire and The Humber-Leeds Bradford Research Ethics Committee (REC reference: 13/YH/0310). Two commercial control human dermal fibroblasts were obtained from ATCC and Sigma-Aldrich, while one in-house control cell line was acquired from an otherwise healthy individual, and subsequently reprogrammed to iPSCs alongside DOA fibroblasts, as previously described.[30] Fibroblasts were nucleofected

with episomal vectors encoding reprogramming factors, followed by clonal iPSC isolation. Presence or absence of the *OPA1* variant was confirmed in iPSCs via Sanger sequencing (primers *OPA*1 ex 14; Table S2). One commercial control iPSC line was obtained from Gibco.

CRISPR-corrected DOA-iPSCs were generated via HDR of *OPA1* c.1334G>A using CRISPR-Cas9 RNP targeting of *OPA1* exon 14 and ssDNA repair templates. CRISPR-Cas9 gRNAs and ssDNA templates were designed using Benchling CRISPR design software. gRNAs were designed to target as close as possible to the 1334G>A point variant in *OPA1* exon 14 (Table S3) and to maximize on-target and minimize off-target activity, according to the algorithms developed by Doench et al.[34] and Hsu et al.[33] Once designed, gRNAs were ordered as two-component CRISPR RNA (crRNA) and trans-activating CRISRPR RNA (tracrRNA; Integrated DNA Technologies, IDT) for downstream assembly. ssDNA HDR templates were designed in accordance with previously optimized specifications[31,32] and ordered as Ultramer oligos chemically modified to include phosphorothioate bonds between the first 3 and last 3 nucleotides. All reagents were purchased from IDT; gRNAs were purchased as crRNAs and tracrRNAs, respectively.

Following their design, *OPA1*-targeting gRNAs were validated through the use of a T7 endonuclease I (T7EI; NEB) assay, which cleaves DNA heteroduplexes, following the manufacturer's instructions. Briefly, DOA-iPSCs were nucleofected with RNPs targeting either OPA1 (*OPA1* gRNA 1 and 2) or non-targeting gRNAs or Cas9 only. 48 h after nucleofection, gDNA was extracted, and the target region surrounding the c.1334G>A mutation was amplified using standard PCR techniques (primers, Table S2) before heteroduplex formation on a thermocycler. 15 μL of heteroduplex product was subsequently treated with 1 μL of T7EI, incubated for 30 min at 37°C. T7EI-treated heteroduplex reactions were resolved on a 2% agarose gel alongside untreated reactions to determine *OPA1* gRNA specificity.

Nucleofection of DOA-iPSCs with *OPA1*-targeting RNP and ssDNA donors was performed following the IDT protocol. Briefly, RNP:gRNA complexes were prepared following IDT guidelines and mixed with 200,000 single-cell DOA-iPSCs in P3 Primary Cell Nucleofector solution (Lonza) containing 4 μM Electroportation Enhancer (IDT). Samples were subsequently nucleofected using a Lonza 4D-nucleofector with X-unit (Lonza), using electroporation program CA-137. Post-nucleofection iPSCs were seeded onto 24-well plates coated with 0.5 μg/cm^2 rhLaminin 521, with or without 30 μM HDRe (IDT), and cultured until passage. iPSCs were passaged twice, first into a 6-well plate for 6 days of culture, followed by a second passage with serial dilution at 4,000, 2,000, 1,000, 500, 250, and 125 cells per well to generate single-cell-derived iPSC colonies. During the second expansion of iPSCs, gDNA was used, in combination with an overlap-PCR-generated template (primers, Table S4), to estimate the efficiency of HDR within the nucleofected cell population via TIDER (http://shinyapps.datacurators.nl/tider/). Individual iPSC colonies were isolated from the condition with the highest HDR frequency and further cultured before gDNA extraction and Sanger sequencing to screen for gene edited clones. Correctly edited colonies were identified by sequence alignment on Benchling.

Finally, off-targets with the highest homology to designed gRNAs were predicted via Off-Spotter (https://cm.jefferson.edu/Off-Spotter/). The top 5 off-targets were expanded by PCR (Table S4) before Sanger sequencing and alignment on Benchling (https://www.benchling.com/) to confirm absence of mutations.

RNA extraction and cDNA synthesis

Total RNA was extracted using the RNeasy Mini Kit (QIAGEN) according to the manufacturer's instructions. First strand cDNA synthesis was completed using the Tetro cDNA synthesis kit (Bioline) using 100 ng total RNA per reaction.

PCR, RT-PCR, and qPCR

PCR amplification of genomic DNA was performed using 2× GoTaq green master mix (Promega) and 0.5 μM forward and reverse primers (Tables S2, S4, and S5), loaded with 100 ng total gDNA. PCR reactions were incubated in a thermocycler for a total of 35 cycles before analysis via gel electrophoresis.

RT-PCR analysis of cDNA was completed using 2× GoTaq green master mix (Promega) and 0.5 μM forward and reverse primers. cDNA was diluted 1:5 with ddH_2O and 5 μL used per reaction. RT-PCR reactions were incubated in a thermocycler for a total of 28–30 cycles.

qPCR was completed using the SYBR Green method run on a Quantstudio 6 Flex real-time PCR system (Thermo Fisher). cDNA samples were diluted to 50 ng RNA/100 μL ddH_2O and 5 μL used per reaction (primers, Table S6), each sample was loaded in triplicate, qPCR data were collected in QuantStudio Real-Time PCR software (Applied Biosystems), and raw data were exported to Microsoft Excel. Data were normalized to the geometric mean of two reference genes, *GAPDH* and *ACTIN*.

Mitochondrial bioenergetic assessment

Live assessment of cellular bioenergetics was performed using the Seahorse XFe96 extracellular flux analyzer (Seahorse Bioscience) following the manufacturer's instructions. Cells were seeded at 30,000 cells onto laminin-coated (3.33 μg/mL) plates 48 h prior to analysis. On the day of experimentation, cell medium was changed to XF base media approximately 60 min before analysis and incubated at 37°C in a CO_2-free incubator. The Seahorse machine was calibrated before beginning measurement of mitochondrial respiration (OCR). After baseline measurements, cells were treated with 1 μM oligomycin, 1 μM FCCP, and 0.5 μM rotenone and antimycin-A. Once completed, cells were lysed with radioimmunoprecitation assay (RIPA) buffer plus 2% proteinase inhibitor cocktail (PIC) and total protein determined with a Pierce BCA Assay Kit (Thermo Fisher). Data were subsequently analyzed using Wave software (Seahorse Bioscience) and raw data exported to Microsoft Excel.

Assessment of mtDNA deletion and copy number

To detect possible mtDNA deletions, LR-PCR was used to target the major arc of the mitochondrial genome, as previously described.[18,19] Total DNA was isolated from cell cultures and 50 ng combined with a 2× master mix consisting of 12.5 μL GoTaq Long PCR (Promega) and 0.2 μM of both forward and reverse primers targeting a 10 kb mtDNA fragment (Table S7). Amplifications were incubated in a thermocycler at the following conditions: 2 min at 95°C; 35 cycles of 95°C for 30 s, 58°C for 30 s, and 68°C for 10 min; and a final extension of 11 min at 68°C. LR-PCR reaction products were analyzed on a 0.7% agarose gel containing SafeView and visualized on BioRad ChemiDoc XRS+.

Quantification of mtDNA copy number, WT, and mtDNA deletion levels was adapted from previous studies.[18] Total DNA was extracted using a Wizard SV Genomic DNA Purification System (Promega) following the manufacturer's instructions. qPCR was completed as above using primers designed to enable quantification of mtDNA genes, *MTND1* and *MTDN4* (Table S8), and nuclear DNA-encoded genes, *B2M* and *GAPDH* (Table S8). Sample DNA was diluted to 10 ng/μL and a total of 50 ng total DNA loaded per reaction. Each sample was loaded in triplicate and run on a Quantstudio 6 Flex Real-Time PCR system, with data collected in QuantStudio Real-Time PCR software. Reaction conditions were as follows: 3 min at 95°C followed by 40 cycles of 10 s at 95°C then 1 min at 62.5°C. Analysis was completed using the ΔΔCt method to calculate WT mtDNA levels, where *MTND4* was the target gene and *MTND1* was the reference gene. *MTND4* levels were first normalized to *MTND1* levels before samples were normalized to WT control levels to determine WT mtDNA percentage. MtDNA copy number was calculated using the following equation: copy number = $2(2^{-\Delta Ct})$, where *MTND1* was the target gene and the geometric mean of *GAPDH* and *B2M* used as reference genes.

Mitochondrial network analysis

Mitochondrial network analysis was based on previously published studies.[36,46] iPSCs were dissociated using TrypLE express and seeded at 10,000/cm^2 in glass-bottomed dishes (Ibidi) or chamber slides (Thermo Fisher) and cultured at 37°C in 5% CO_2 for 24 h. Subsequently, iPSCs were fixed in 4% paraformaldehyde for 10 min at room temperature, permeabilized in 0.1% Triton X-100 for 10 min at room temperature, and blocked for 1 h in 3% bovine serum albumin and 10% normal donkey serum dissolved in Dulbecco's phosphate-buffered saline (DPBS). Mitochondrial networks were subsequently stained using a primary antibody targeting TOMM20 (Santa Cruz) and incubated at 4°C overnight, followed by incubation with a donkey anti-mouse Alexa Fluor 488 or 555 secondary antibody, incubated at room temperature for 2 h. Finally, nuclei were identified using DAPI (2 μ/mL) and mounted using Vectashield Vibrance mounting media (Vector Labs).

Confocal imaging for fragmentation analysis was completed using a Leica SP8 inverted Laser Scanning Confocal Microscope (Leica Microsystems), with a 63× oil immersion lens and automated image deconvolution through Huygens software (Scientific Volume Imaging). Quantification was completed in at least 30 cells from 3 individual cell preparations using an image pre-processing pipeline[46] and the MiNa toolset.[36] After image acquisition, cells were individually cropped and pre-processed by using ImageJ functions: Subtract background (Radius 50 pixels), Sigma filter plus (4, 2, 0.2), CLAHE (64, 256, 2), median filtering (radius = 10), make binary, despeckle, and remove outliers (2, 50). MiNa raw data, including network composition (number of branched networks and number of individuals, mitochondria without branches), mean network size (number of branches per network), and mean network branch length (μm), were transferred to Microsoft Excel before analysis.

Flow cytometry analysis of iPSC apoptosis and TMRE incorporation

Analysis of iPSC susceptibility to apoptotic stimuli was carried out using Annexin V-FITC (fluorescein isothiocyanate) and PI (BioLegend), according to the manufacturer's instructions. Briefly, iPSCs were seeded as single cells at a density of 25,000/cm^2. 24 h after seeding, cells were treated with 10 nM staurosporine (Sigma) or DMSO for either 8 or 24 h at 37°C in 5% CO_2. iPSCs were then dissociated with TrypLE express for 15 min, pelleted at 300 × *g*, and subsequently resuspended in binding buffer with 5 μL Annexin V-FITC and 5 μL PI. Cells were incubated in the dark at room temperature. Following staining, cells were washed with binding buffer and analyzed on a BD LSR Fortessa X-20 (BD). FlowJo software (FlowJo) was used for analysis and illustration of flow cytometry data, with population gates determined using unstained and single-stained positive control samples to determine positive and negative populations for either Annexin V or PI before application of gates to experimental samples.

LDH cytotoxicity assay

Quantification of cellular LDH release was completed using the Cytotoxicity Detection Kit Plus (Roche) following the manufacturer's guidelines. Briefly, iPSCs were seeded as single cells at a density of 25,000 cells per well of a 96-well plate. 24 h after seeding, iPSCs were treated with 10 nM staurosporine or DMSO vehicle and incubated for a further 24 h at 37°C in 5% CO_2. Following treatment, iPSCs were centrifuged at 300 × *g* for 5 min before 100 μL of supernatant was transferred to a fresh 96-well plate. Supernatants were subsequently incubated with 100 μL of reaction mixture for 30 min at room temperature, before reactions were stopped using 50 μL stop solution. Reactions were quantified using a Safire Microplate reader (Tecan), using absorbance wavelength of 492 nm and reference wavelength 630 nm. Staurosporine cytotoxicity was calculated as follows: cytotoxicity % = (experimental value − low control)/(high control − low control) × 100, where low control samples are iPSCs treated with DMSO and high control samples are iPSCs lysed with 1% Triton X-100 1 h before quantification.

Statistical analysis

Statistical analysis was completed using GraphPad Prism 6 software (GraphPad) using a one-way AVONA with a Tukey's multiple

comparison test. Results are represented as arithmetic mean ± standard error of the mean (SEM) unless otherwise stated.

SUPPLEMENTAL INFORMATION

Supplemental information can be found online at https://doi.org/10.1016/j.omtn.2021.08.015.

ACKNOWLEDGMENTS

P.Y.-W.-M. is supported by a Clinician Scientist Fellowship Award (G1002570) from the Medical Research Council (UK). This work was supported by funding from Moorfields Eye Charity, Fight for Sight (UK), the Wellcome Trust (205041), the MRC (UK), the Isaac Newton Trust (UK), the Addenbrooke's Charitable Trust, the National Eye Research Centre (UK), the UK National Institute of Health Research (NIHR) as part of the Rare Diseases Translational Research Collaboration, and the NIHR Biomedical Research Centre based at Moorfields Eye Hospital NHS Foundation Trust and UCL Institute of Ophthalmology. The views expressed are those of the authors and not necessarily those of the NHS, the NIHR, or the Department of Health.

AUTHOR CONTRIBUTIONS

P.E.S. designed and performed experiments, analyzed data, and wrote the manuscript draft. P.R.L.P designed experiments and edited the manuscript. G.S. and T.N. assisted with experiments. J.v.d.S. and J.P.C. edited the manuscript. P.Y.-W.-M. and M.E.C. directed the study design, secured funding support, and edited the manuscript. All authors approved the final manuscript.

DECLARATION OF INTERESTS

The authors declare no competing interests.

REFERENCES

1. Yu-Wai-Man, P., and Chinnery, P.F. (2013). Dominant optic atrophy: novel OPA1 mutations and revised prevalence estimates. Ophthalmology *120*, 1712–1712.e1.

2. Lenaers, G., Hamel, C., Delettre, C., Amati-Bonneau, P., Procaccio, V., Bonneau, D., Reynier, P., and Milea, D. (2012). Dominant optic atrophy. Orphanet J. Rare Dis. *7*, 46.

3. Yu-Wai-Man, P., Bailie, M., Atawan, A., Chinnery, P.F., and Griffiths, P.G. (2011). Pattern of retinal ganglion cell loss in dominant optic atrophy due to OPA1 mutations. Eye (Lond.) *25*, 596–602.

4. Ferré, M., Caignard, A., Milea, D., Leruez, S., Cassereau, J., Chevrollier, A., Amati-Bonneau, P., Verny, C., Bonneau, D., Procaccio, V., and Reynier, P.; Improved Locus-Specific Database for OPA1 Mutations Allows Inclusion of Advanced Clinical Data (2015). Improved locus-specific database for OPA1 mutations allows inclusion of advanced clinical data. Hum. Mutat. *36*, 20–25.

5. Yu-Wai-Man, P., Griffiths, P.G., Gorman, G.S., Lourenco, C.M., Wright, A.F., Auer-Grumbach, M., Toscano, A., Musumeci, O., Valentino, M.L., Caporali, L., et al. (2010). Multi-system neurological disease is common in patients with OPA1 mutations. Brain *133*, 771–786.

6. Davies, V.J., Hollins, A.J., Piechota, M.J., Yip, W., Davies, J.R., White, K.E., Nicols, P.P., Boulton, M.E., and Votruba, M. (2007). Opa1 deficiency in a mouse model of autosomal dominant optic atrophy impairs mitochondrial morphology, optic nerve structure and visual function. Hum. Mol. Genet. *16*, 1307–1318.

7. Song, Z., Chen, H., Fiket, M., Alexander, C., and Chan, D.C. (2007). OPA1 processing controls mitochondrial fusion and is regulated by mRNA splicing, membrane potential, and Yme1L. J. Cell Biol. *178*, 749–755.

8. Amati-Bonneau, P., Guichet, A., Olichon, A., Chevrollier, A., Viala, F., Miot, S., Ayuso, C., Odent, S., Arrouet, C., Verny, C., et al. (2005). OPA1 R445H mutation in optic atrophy associated with sensorineural deafness. Ann. Neurol. *58*, 958–963.

9. Zanna, C., Ghelli, A., Porcelli, A.M., Karbowski, M., Youle, R.J., Schimpf, S., Wissinger, B., Pinti, M., Cossarizza, A., Vidoni, S., et al. (2008). OPA1 mutations associated with dominant optic atrophy impair oxidative phosphorylation and mitochondrial fusion. Brain *131*, 352–367.

10. Alavi, M.V., Bette, S., Schimpf, S., Schuettauf, F., Schraermeyer, U., Wehrl, H.F., Ruttiger, L., Beck, S.C., Tonagel, F., Pichler, B.J., et al. (2007). A splice site mutation in the murine Opa1 gene features pathology of autosomal dominant optic atrophy. Brain *130*, 1029–1042.

11. Suárez-Rivero, J.M., Villanueva-Paz, M., de la Cruz-Ojeda, P., de la Mata, M., Cotán, D., Oropesa-Ávila, M., de Lavera, I., Álvarez-Córdoba, M., Luzón-Hidalgo, R.S.-A., and Sánchez-Alcázar, J.A. (2017). Mitochondrial Dynamics in Mitochondrial Diseases. Diseases *5*, 1.

12. Kushnareva, Y.E., Gerencser, A.A., Bossy, B., Ju, W.-K., White, A.D., Waggoner, J., Ellisman, M.H., Perkins, G., and Bossy-Wetzel, E. (2013). Loss of OPA1 disturbs cellular calcium homeostasis and sensitizes for excitotoxicity. Cell Death Differ. *20*, 353–365.

13. Del Dotto, V., Mishra, P., Vidoni, S., Fogazza, M., Maresca, A., Caporali, L., McCaffery, J.M., Cappelletti, M., Baruffini, E., Lenaers, G., et al.; OPA1 Isoforms in the Hierarchical Organization of Mitochondrial Functions (2017). OPA1 Isoforms in the Hierarchical Organization of Mitochondrial Functions. Cell Rep. *19*, 2557–2571.

14. Yang, L., Tang, H., Lin, X., Wu, Y., Zeng, S., Pan, Y., Li, Y., Xiang, G., Lin, Y.-F., Zhuang, S.-M., et al. (2020). OPA1-Exon4b Binds to mtDNA D-Loop for Transcriptional and Metabolic Modulation, Independent of Mitochondrial Fusion. Front. Cell Dev. Biol. *8*, 180.

15. Kim, J.Y., Hwang, J.-M., Ko, H.S., Seong, M.-W., Park, B.-J., and Park, S.S. (2005). Mitochondrial DNA content is decreased in autosomal dominant optic atrophy. Neurology *64*, 966–972.

16. Chen, L., Liu, T., Tran, A., Lu, X., Tomilov, A.A., Davies, V., Cortopassi, G., Chiamvimonvat, N., Bers, D.M., Votruba, M., and Knowlton, A.A. (2012). OPA1 mutation and late-onset cardiomyopathy: mitochondrial dysfunction and mtDNA instability. J. Am. Heart Assoc. *1*, e003012.

17. Amati-Bonneau, P., Valentino, M.L., Reynier, P., Gallardo, M.E., Bornstein, B., Boissière, A., Campos, Y., Rivera, H., de la Aleja, J.G., Carroccia, R., et al. (2008). OPA1 mutations induce mitochondrial DNA instability and optic atrophy 'plus' phenotypes. Brain *131*, 338–351.

18. Yu-Wai-Man, P., Sitarz, K.S., Samuels, D.C., Griffiths, P.G., Reeve, A.K., Bindoff, L.A., Horvath, R., and Chinnery, P.F. (2010). OPA1 mutations cause cytochrome c oxidase deficiency due to loss of wild-type mtDNA molecules. Hum. Mol. Genet. *19*, 3043–3052.

19. Hudson, G., Amati-Bonneau, P., Blakely, E.L., Stewart, J.D., He, L., Schaefer, A.M., Griffiths, P.G., Ahlqvist, K., Suomalainen, A., Reynier, P., et al. (2008). Mutation of OPA1 causes dominant optic atrophy with external ophthalmoplegia, ataxia, deafness and multiple mitochondrial DNA deletions: a novel disorder of mtDNA maintenance. Brain *131*, 329–337.

20. Caglayan, S., Hashim, A., Cieslar-Pobuda, A., Jensen, V., Behringer, S., Talug, B., Chu, D.T., Pecquet, C., Rogne, M., Brech, A., et al. (2020). Optic Atrophy 1 Controls Human Neuronal Development by Preventing Aberrant Nuclear DNA Methylation. iScience *23*, 101154.

21. Klopstock, T., Metz, G., Yu-Wai-Man, P., Büchner, B., Gallenmüller, C., Bailie, M., Nwali, N., Griffiths, P.G., von Livonius, B., Reznicek, L., et al. (2013). Persistence of the treatment effect of idebenone in Leber's hereditary optic neuropathy. Brain *136*, e230.

22. Romagnoli, M., La Morgia, C., Carbonelli, M., Di Vito, L., Amore, G., Zenesini, C., Cascavilla, M.L., Barboni, P., and Carelli, V. (2020). Idebenone increases chance of stabilization/recovery of visual acuity in OPA1-dominant optic atrophy. Ann. Clin. Transl. Neurol. *7*, 590–594.

23. Smith, T.G., Seto, S., Ganne, P., and Votruba, M. (2016). A randomized, placebo-controlled trial of the benzoquinone idebenone in a mouse model of OPA1-related dominant optic atrophy reveals a limited therapeutic effect on retinal ganglion cell dendropathy and visual function. Neuroscience *319*, 92–106.
24. Schimpf, S., Fuhrmann, N., Schaich, S., and Wissinger, B. (2008). Comprehensive cDNA study and quantitative transcript analysis of mutant OPA1 transcripts containing premature termination codons. Hum. Mutat. *29*, 106–112.
25. Sarzi, E., Seveno, M., Piro-Mégy, C., Elzière, L., Quilès, M., Péquignot, M., Müller, A., Hamel, C.P., Lenaers, G., and Delettre, C. (2018). OPA1 gene therapy prevents retinal ganglion cell loss in a Dominant Optic Atrophy mouse model. Sci. Rep. *8*, 2468.
26. Cipolat, S., Martins de Brito, O., Dal Zilio, B., and Scorrano, L. (2004). OPA1 requires mitofusin 1 to promote mitochondrial fusion. Proc. Natl. Acad. Sci. USA *101*, 15927–15932.
27. Yu-Wai-Man, P., Newman, N.J., Carelli, V., Moster, M.L., Biousse, V., Sadun, A.A., Klopstock, T., Vignal-Clermont, C., Sergott, R.C., Rudolph, G., et al. (2020). Bilateral visual improvement with unilateral gene therapy injection for Leber hereditary optic neuropathy. Sci. Transl. Med. *12*, eaaz7423.
28. Newman, N.J., Yu-Wai-Man, P., Carelli, V., Moster, M.L., Biousse, V., Vignal-Clermont, C., Sergott, R.C., Klopstock, T., Sadun, A.A., Barboni, P., et al.; LHON Study Group (2021). Efficacy and Safety of Intravitreal Gene Therapy for Leber Hereditary Optic Neuropathy Treated within 6 Months of Disease Onset. Ophthalmology *128*, 649–660.
29. Anzalone, A.V., Koblan, L.W., and Liu, D.R. (2020). Genome editing with CRISPR-Cas nucleases, base editors, transposases and prime editors. Nat. Biotechnol. *38*, 824–844.
30. Schwarz, N., Carr, A.-J., Lane, A., Moeller, F., Chen, L.L., Aguilà, M., Nommiste, B., Muthiah, M.N., Kanuga, N., Wolfrum, U., et al. (2015). Translational read-through of the RP2 Arg120stop mutation in patient iPSC-derived retinal pigment epithelium cells. Hum. Mol. Genet. *24*, 972–986.
31. Richardson, C.D., Ray, G.J., DeWitt, M.A., Curie, G.L., and Corn, J.E. (2016). Enhancing homology-directed genome editing by catalytically active and inactive CRISPR-Cas9 using asymmetric donor DNA. Nat. Biotechnol. *34*, 339–344.
32. Paquet, D., Kwart, D., Chen, A., Sproul, A., Jacob, S., Teo, S., Olsen, K.M., Gregg, A., Noggle, S., and Tessier-Lavigne, M. (2016). Efficient introduction of specific homozygous and heterozygous mutations using CRISPR/Cas9. Nature *533*, 125–129.
33. Hsu, P.D., Scott, D.A., Weinstein, J.A., Ran, F.A., Konermann, S., Agarwala, V., Li, Y., Fine, E.J., Wu, X., Shalem, O., et al. (2013). DNA targeting specificity of RNA-guided Cas9 nucleases. Nat. Biotechnol. *31*, 827–832.
34. Doench, J.G., Fusi, N., Sullender, M., Hegde, M., Vaimberg, E.W., Donovan, K.F., Smith, I., Tothova, Z., Wilen, C., Orchard, R., et al. (2016). Optimized sgRNA design to maximize activity and minimize off-target effects of CRISPR-Cas9. Nat. Biotechnol. *34*, 184–191.
35. Brinkman, E.K., and van Steensel, B. (2019). Rapid Quantitative Evaluation of CRISPR Genome Editing by TIDE and TIDER. In CRISPR Gene Editing: Methods and Protocols, Y. Luo, ed. (New York, NY: Springer New York), pp. 29–44.
36. Valente, A.J., Maddalena, L.A., Robb, E.L., Moradi, F., and Stuart, J.A. (2017). A simple ImageJ macro tool for analyzing mitochondrial network morphology in mammalian cell culture. Acta Histochem. *119*, 315–326.
37. Poyton, R.O., and McEwen, J.E. (1996). Crosstalk between nuclear and mitochondrial genomes. Annu. Rev. Biochem. *65*, 563–607.
38. Cox, D.B.T., Platt, R.J., and Zhang, F. (2015). Therapeutic genome editing: prospects and challenges. Nat. Med. *21*, 121–131.
39. Holt, I.J., He, J., Mao, C.-C., Boyd-Kirkup, J.D., Martinsson, P., Sembongi, H., Reyes, A., and Spelbrink, J.N. (2007). Mammalian mitochondrial nucleoids: organizing an independently minded genome. Mitochondrion *7*, 311–321.
40. Bender, A., Krishnan, K.J., Morris, C.M., Taylor, G.A., Reeve, A.K., Perry, R.H., Jaros, E., Hersheson, J.S., Betts, J., Klopstock, T., et al. (2006). High levels of mitochondrial DNA deletions in substantia nigra neurons in aging and Parkinson disease. Nat. Genet. *38*, 515–517.
41. Yu-Wai-Man, P., Turnbull, D.M., and Chinnery, P.F. (2002). Leber hereditary optic neuropathy. J. Med. Genet. *39*, 162–169.
42. Gammage, P.A., Moraes, C.T., and Minczuk, M. (2018). Mitochondrial Genome Engineering: The Revolution May Not Be CRISPR-Ized. Trends Genet. *34*, 101–110.
43. Mok, B.Y., de Moraes, M.H., Zeng, J., Bosch, D.E., Kotrys, A.V., Raguram, A., Hsu, F., Radey, M.C., Peterson, S.B., Mootha, V.K., et al. (2020). A bacterial cytidine deaminase toxin enables CRISPR-free mitochondrial base editing. Nature *583*, 631–637.
44. Howlett, E.H., Jensen, N., Belmonte, F., Zafar, F., Hu, X., Kluss, J., Schüle, B., Kaufman, B.A., Greenamyre, J.T., and Sanders, L.H. (2017). LRRK2 G2019S-induced mitochondrial DNA damage is LRRK2 kinase dependent and inhibition restores mtDNA integrity in Parkinson's disease. Hum. Mol. Genet. *26*, 4340–4351.
45. Sanders, L.H., Laganière, J., Cooper, O., Mak, S.K., Vu, B.J., Huang, Y.A., Paschon, D.E., Vangipuram, M., Sundararajan, R., Urnov, F.D., et al. (2014). LRRK2 mutations cause mitochondrial DNA damage in iPSC-derived neural cells from Parkinson's disease patients: reversal by gene correction. Neurobiol. Dis. *62*, 381–386.
46. Chaudhry, A., Shi, R., and Luciani, D.S. (2020). A pipeline for multidimensional confocal analysis of mitochondrial morphology, function, and dynamics in pancreatic β-cells. Am. J. Physiol. Endocrinol. Metab. *318*, E87–E101.

OMTN, Volume *26*

Supplemental information

CRISPR-Cas9 correction of *OPA1* c.1334G>A: p.R445H restores mitochondrial homeostasis in dominant optic atrophy patient-derived iPSCs

Paul E. Sladen, Pedro R.L. Perdigão, Grace Salsbury, Tatiana Novoselova, Jacqueline van der Spuy, J. Paul Chapple, Patrick Yu-Wai-Man, and Michael E. Cheetham

Supplementary material

CRISPR/Cas9 correction of *OPA1* c.1334G>A: p.R445H restores mitochondrial homeostasis in Dominant Optic Atrophy patient-derived iPSCs.

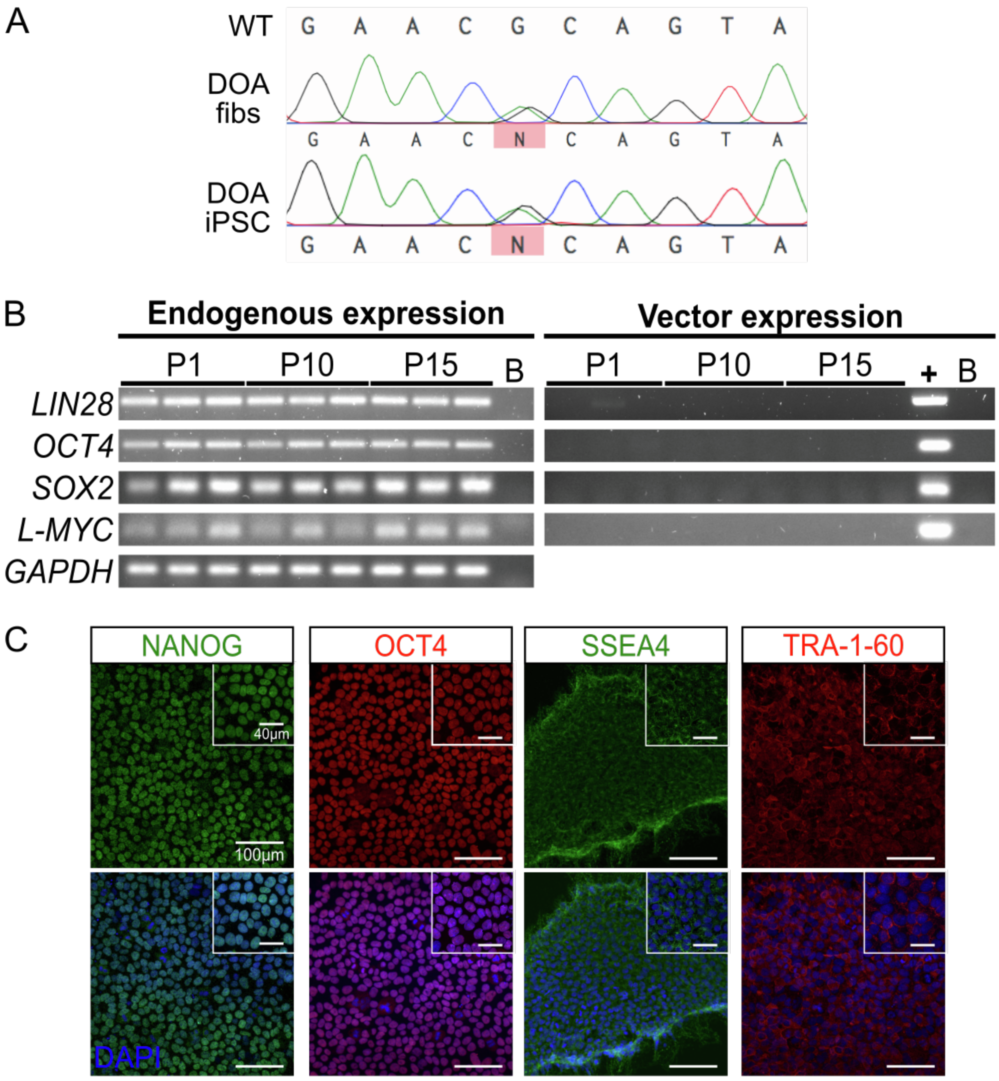

Figure S1. Generation of iPSCs carrying an *OPA1* c.1334G>A variant. Patient-derived fibroblasts carrying a c.1334G>A variant in *OPA1* were reprogrammed to iPSC by nucleofection of episomal plasmids.
(A) Post-reprogramming Sanger sequencing confirmed the presence of the G>A (N base highlighted in red) mutation within both patient-derived fibroblasts and iPSCs.
(B) RT-PCR analysis confirmed the expression of genes associated with endogenous self-renewal, including *LIN28*, *OCT4*, *SOX2*, and *L-MYC*, in DOA-iPSC. Similarly, there was negligible expression of episomal plasmid (vector) associated genes across all passage (P1, P10 and P15) of assessed iPSCs. *GAPDH* cDNA loading control. + = episomal plasmid control, B = no cDNA.
(C) Immunofluorescent staining of DOA-iPSC confirmed expression of embryonic stem cell associated proteins NANOG, OCT4, SSEA4 and TRA-160. Scale bars 100 µm and 40 µm for inset.

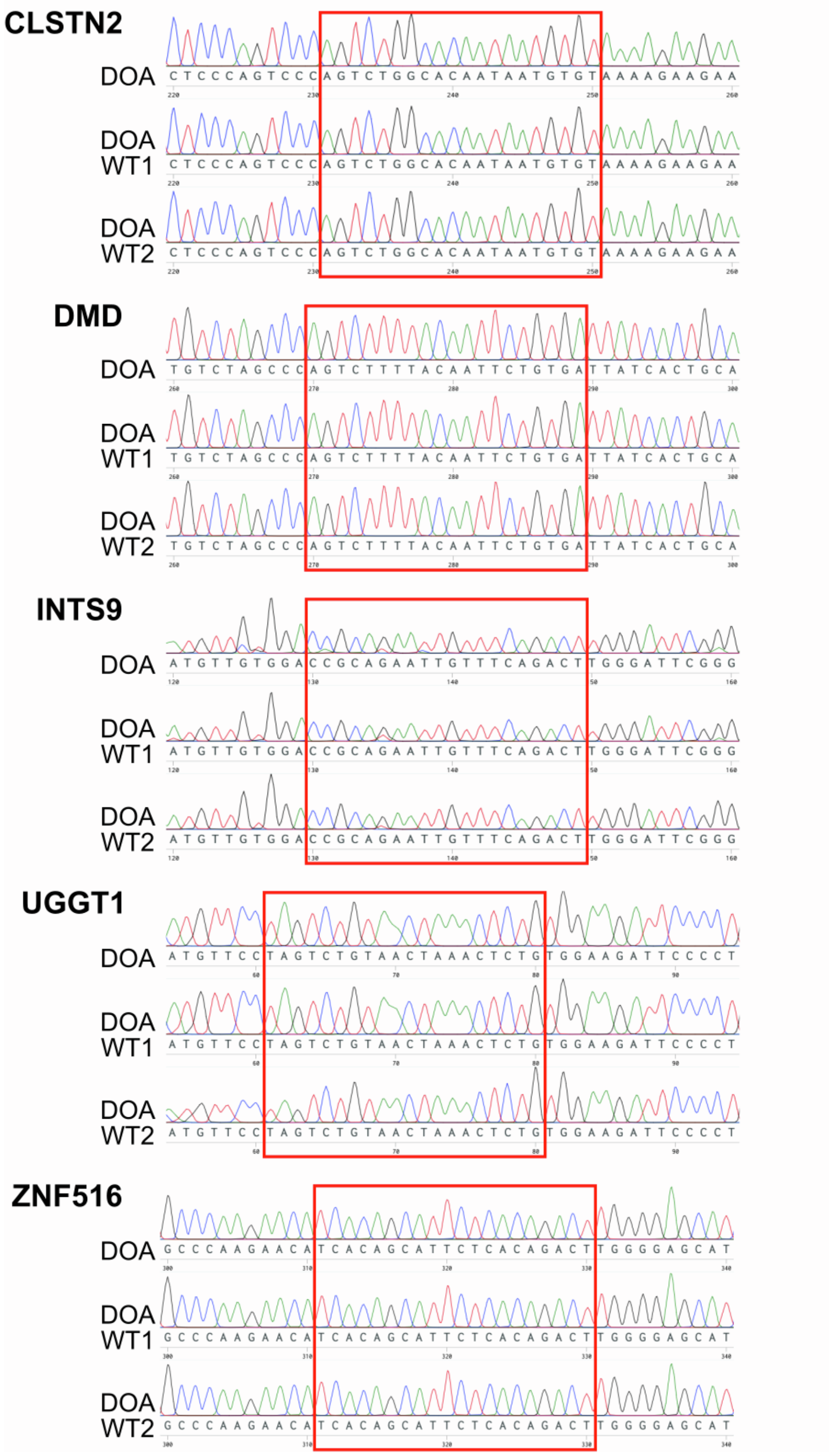

Figure S2. CRISPR/Cas9 off-target sequencing.
The top 5 predicted CRISPR/Cas9 off-target sites were determined using Off-spotter. Targets were amplified by PCR for both DOA- and corrected WT1 and WT2 iPSCs, followed by Sanger sequencing and alignment on Benchling. No mutations were found in either corrected cell line.

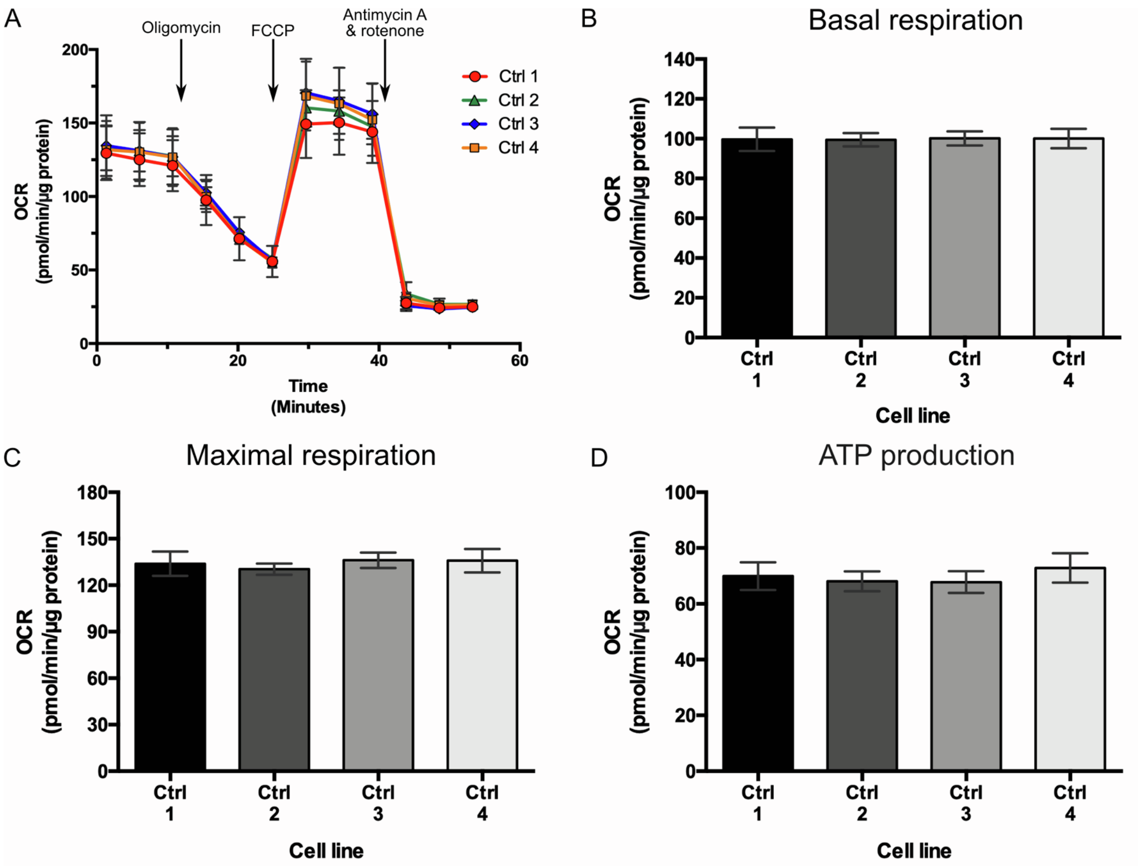

Figure S3. Comparison of mitochondrial bioenergetics for 4 independent WT iPSC lines. **(A)** Seahorse oxygen consumption rate (OCR) profiles were generated using the Seahorse XFe96 Analyzer for four independent control (ctrl) iPSC lines. 1µM oligomycin, 1µM FCCP and 0.5 µM antimycin A and 0.5 µM rotenone were injected at designated time points. Symbols represent mean OCR ± SEM.

(B-D) Analysis of bioenergetic profiles reveals the respiratory phenotype of iPSC lines. No significant differences were detected for the four independent iPSC lines for basal respiration **(B)**, maximal respiration **(C)** and ATP production **(D)**. N=34-48 replicates per cell line, from 3 separate experiments.

Table S1. Top 5 predicted off-target genes for 1334G>A gRNA 2.

The top 5 off-target genes were determined on Off-Spotter and amplified by PCR before Sanger sequencing and sequence alignment. No off-target CRISPR/Cas9 induced mutations were identified.

Gene	Chromosome	Position	gRNA mismatches	CRISPR-induced mutation
CLSTN2	3	Intronic	3	No
DMD	X	Intronic	5	No
INTS9	8	Intronic	4	No
UGGT1	2	Intronic	3	No
ZNF516	18	Intronic	5	No

Table S2. Primers used for sequencing DOA iPSCs and generation of TIDER reference template by overlap PCR.

Gene	Primer		Product size
	Forward	Reverse	
OPA1 ex 14	GCATTTTTGCCTTCTCTTCGT	TGCTTTCCTTTTGGGAGTATG	732
OPA1 ex 14 gRNA 1 C	-	GCGTTCAGCATCGACAGATCCATC	268
OPA1 ex 14 gRNA 1 D	GATGGATCTGTCGATGCTGAACGCAGTATTGTTAC	-	488
OPA1 ex 14 gRNA 2 C	-	GTCCATTTGACTGACAAGGTCTGTAACAATACTGCGTTCAGCATCCACAGATCC	301
OPA1 ex 14 gRNA 2 D	TTGTTACAGACCTTGTCAGTCAAATGGAC	-	260

Table S3. gRNAs designed for targeting OPA1 exon 14.

gRNA	Guide oligonucleotide sequence	GC (%)	Distance from mutation (bp)	Predicted off-targets (Off-spotter)
gRNA 1	ATTTTTTTCAGATGGATCTG	30	65	618
gRNA 2	ACACAGTATTGTTACAGACT	35	44	314

Table S4. CRISPR/Cas9 gRNA off-target primers.

Gene	Primer		Product size
	Forward	Reverse	
CLTSN2	CTTGCCTTACAGGGTTGTGGT	GCCTCCCTGACATGACTTCTC	569
DMD	ACCATCTTCATTCCCTCTCCA	AGCCTCCACGTAAGTTGCATT	511
INTS9	AACCTGTAGACCCCTTTGCC	TCTGTGAGGGAGGAAGAAGCC	501
UGGT1	ACATGCTAACAGATGCCCAG	TCCCAACTCCCACTGAACAA	458
ZNF516	TAGGTGTGTAGGCGATGGTC	TCCTCCTGTACTAAGTGTGCAA	628

Table S5. Primers used for analysis of endogenous and vector-encoded self-renewal markers by RT-PCR.

Gene	Primer		Product size
	Forward	Reverse	
LIN29 CDS	AGCCATATGGTAGCCTCATGTCCGC	TCAATTCTGTGCCTCCGGGAGCAGGGTAGG	129
LIN28 plasmid	AGCCATATGGTAGCCTCATGTCCGC	TAGCGTAAAAGGAGCAACATAG	251
L-MYC CDS	GCGAACCCAAGACCCAGGCCTGCTCC	CAGGGGGTCTGCTCGCACCGTGATG	143
L-MYC plasmid	GGCTGAGAAGAGGATGGCTAC	TTTGTTTGACAGGAGCGACAAT	122
OCT4 CDS	CCCCAGGGCCCCATTTTGGTACC	ACCTCAGTTTGAATGCATGGGAGAGC	143
OCT4 plasmid	CATTCAAACTGAGGTAAGGG	TAGCGTAAAAGGAGCAACATAG	124
SOX2 CDS	TTCACATGTCCCAGCACTACCAGA	TCACATGTGTGAGAGGGGCAGTGTGC	80
SOX2 plasmid	TTCACATGTCCCAGCACTACCAGA	TTTGTTTGACAGGAGCGACAAT	111

Table S6. Primers used for qPCR quantification of *OPA1* expression.

Gene	Primer		Product size
	Forward	Reverse	
ACTIN	CCAACCGCGAGAAGATGA	CCAGAGGCGTACAGGGATAG	97
GAPDH	CCCCACCACACTGAATCTCC	GGTACTTTATTGATGGTACATGACAAG	105
OPA1	CGACCCCAATTAAGGACATCC	GCGAGGCTGGTAGCCATATTT	102

Table S7. Primers used to expand mtDNA via LR-PCR.

Gene	Primer		Product size
	Forward	Reverse	
mtDNA 10kb	CCCTCTCTCCTACTCCTG	CAGGTGGTCAAGTATTTATGG	9932

Table S8. Primers used to for mtDNA quantification by qPCR.

Gene	Primer		Product size
	Forward	Reverse	
B2M	CACTGAAAAAGATGAGTATGCC	AACATTCCCTGACAATCCC	231
GAPDH	CTCATCCAAGACTGGCTCCTCC	CAGCGTACTCCCCACATCAC	243
MTND1	ACGCCATAAAACTCTTCACCAAAG	GGGTTCATAGTAGAAGAGCGATGG	111
MTND4	ACCTTGGCTATCATCACCCGAT	AGTGCGATGAGTAGGGGAAGG	107
